# Stevens-Johnson syndrome and toxic epidermal necrolysis associated with immune checkpoint inhibitors: a systematic review

**DOI:** 10.3389/fimmu.2024.1414136

**Published:** 2024-07-12

**Authors:** Jia Zhou, Chuan-Peng Wang, Jun Li, Han-Lin Zhang, Chun-Xia He

**Affiliations:** ^1^ Department of Dermatology, State Key Laboratory of Complex Severe and Rare Diseases, Peking Union Medical College Hospital, Chinese Academy of Medical Sciences and Peking Union Medical College, National Clinical Research Center for Dermatologic and Immunologic Diseases, Beijing, China; ^2^ Department of Nephrology, Peking Union Medical College Hospital, Chinese Academy of Medical Sciences and Peking Union Medical College, Beijing, China

**Keywords:** immune checkpoint inhibitor, immune-related adverse events, irAE, severe cutaneous adverse reaction, Stevens-Johnson syndrome, toxic epidermal necrolysis

## Abstract

**Introduction:**

Stevens-Johnson syndrome (SJS) and toxic epidermal necrolysis (TEN) are rare yet life-threatening adverse events associated with immune checkpoint inhibitors (ICIs). This systematic review synthesizes the current literature to elucidate the clinical characteristics and outcomes of patients with ICI-related SJS/TEN.

**Methods:**

We conducted a thorough search across databases including Embase, Web of Science, Cochrane, MEDLINE, Scopus, and PubMed. Selection criteria focused on reports of SJS/TEN among cancer patients treated with ICIs, analyzing clinical manifestations, therapeutic interventions, and outcomes.

**Results:**

Our analysis included 47 articles involving 50 patients with ICI-related SJS/TEN. The cohort had a mean age of 63 years, with a slight male predominance (54%). Most patients had melanoma or non-small cell lung cancer. SJS/TEN typically occurred early, with a median onset of 23 days post-ICI initiation. Treatment primarily involved systemic corticosteroids and intravenous immunoglobulins. The overall mortality rate was 20%, higher for TEN at 32%, with infections and tumor progression as leading causes. Median time from onset to death was 28 days. Survivors experienced a median re-epithelization time of 30 days, positively correlated with the extent of epidermal detachment (r_s_ = 0.639, *p* = 0.009). Deceased patients exhibited a significantly higher proportion of TEN (90% vs. 48%, *p* = 0.029) and a larger epidermal detachment area (90% vs. 30% of the body surface area [BSA], *p* = 0.005) compared to survivors. The combination therapy group showed a higher proportion of TEN compared to corticosteroid monotherapy or non-corticosteroid therapy groups (72% vs. 29% and 50%, *p* = 0.01), with no significant differences in mortality or re-epithelization time. Dual ICI therapy resulted in a higher TEN rate than single therapy (100% vs. 50%, *p* = 0.028). Among single ICI therapies, the sintilimab-treated group trended towards a higher TEN rate (75% vs. 40-50%, *p* = 0.417), a larger detachment area (90% vs. 30-48% of BSA, *p* = 0.172), and a longer re-epithelization time (44 vs. 14-28 days, *p* = 0.036) compared to other ICI groups, while mortality rates remained similar.

**Conclusion:**

ICI-related SJS/TEN substantially impacts patient outcomes. Prospective clinical trials are critically needed to further clarify the pathogenesis and optimize therapeutic regimens.

## 1 Introduction

Immune checkpoint inhibitors (ICIs) have markedly improved outcomes in advanced malignancies, particularly melanoma and non-small cell lung cancer, representing a paradigm shift in their management (1, 2). Nonetheless, the broad activation of the immune system by ICIs has precipitated a spectrum of immune-related adverse events (irAEs), with cutaneous irAEs occurring in 30-50% of treated individuals (3, 4). While most cutaneous irAEs are manageable, Stevens-Johnson syndrome (SJS) and toxic epidermal necrolysis (TEN) present as rare yet potentially fatal reactions, characterized by sudden widespread erythemas and skin peeling, often with accompanying mucositis and fever (5, 6). This disease continuum is classified into three types based on the extent of epidermal detachment: SJS affects less than 10% of the body surface area (BSA); TEN involves more than 30% of the BSA; and SJS/TEN overlap syndrome is defined by 10-30% of BSA involvement (5).

SJS/TEN has been reported with treatments including anti-programmed-death-1 (PD-1), anti-programmed cell death-ligand 1 (PD-L1), anti-cytotoxic T lymphocyte-associated antigen 4 (CTLA-4), and dual PD-1/CTLA-4 blockade (7, 8). ICI-related SJS/TEN can be fatal, primarily due to systemic infection, substantial hemorrhage, and organ failure (7). The National Comprehensive Cancer Network (NCCN) and the European Society for Medical Oncology (ESMO) recommend permanently discontinuing ICIs, using corticosteroids and intravenous immunoglobulins (IVIG), alongside comprehensive supportive care for managing this condition (9, 10). However, these guidelines are founded more upon expert consensus than on evidence-based medicine. A meticulous and comprehensive review delineating the clinical characteristics and treatment outcomes for this patient population is lacking.

This systematic review aims to synthesize and critically evaluate the current literature on ICI-related SJS/TEN, shedding light on the clinical features and prognoses. Our goal is to provide evidence foundation that will inform future research, enhance clinical practices, and contribute to the development of refined management strategies.

## 2 Methods

This systematic review was conducted and reported in accordance with the Preferred Reporting Items for Systematic Reviews and Meta-Analyses (PRISMA) statement (11) and meta-analyses of observational studies in epidemiology (MOOSE) guidelines (12).

### 2.1 Search strategy

An exhaustive search was performed by two independent reviewers (JZ and CPW) on March 14, 2024, utilizing the following databases: Embase, Web of Science, Cochrane, MEDLINE, Scopus, and PubMed. Detailed search terms and strategies are provided as **Supplementary Table 1**. Additionally, we conducted reference list searches of included studies and relevant review articles to retrieve all eligible studies.

### 2.2 Study selection and inclusion criteria

Two reviewers (JZ and CPW) independently scrutinized the results, applying rigorous screening to titles, keywords, and abstracts before assessing full texts for relevance and inclusion criteria. Publications in languages other than English and unpublished conference abstracts were excluded. Articles were also omitted for reporting inaccurate outcomes, missing crucial data, or generalized cohort observations without individual patient details.

Patients were included if they developed SJS/TEN post-ICI therapy, adhering to the classification criteria established by Bastuji-Garin et al. (5), which include: widespread blistering on erythematous skin, mucosal involvement, and histopathological evidence of extensive keratinocyte death or epidermal necrosis with minimal inflammatory infiltration. A possible, probable, or definite association between ICI and SJS/TEN was assessed using the algorithm of drug causality for epidermal necrolysis (ALDEN) scoring system (13).

Any discrepancies arising during the selection process were resolved through consensus, or if necessary, by consulting a third reviewer (CXH).

### 2.3 Data extraction and quality assessment

Two authors (JZ and CPW) independently reviewed the full texts of articles considered potentially eligible, extracted the data onto an electronic form, and a third author (CXH) verified the information. The data included: (1) baseline characteristics such as age, gender, tumor types, ICI regimens, concurrent treatments, and accompanying irAEs; (2) clinical presentations, including the time to SJS/TEN onset, epidermal detachment area, fever or mucositis, histopathologic findings, and severity-of-illness score for toxic epidermal necrolysis (SCORTEN) (14); (3) management and outcomes of SJS/TEN, detailing the dosage and duration of corticosteroids and second-line treatments, acute mortality rate, time to death or re-epithelization, and objective tumor response to ICIs. Efforts to address missing data involved outreach to original authors and application of strategies by the Cochrane Handbook for Systematic Reviews of Interventions (15). Any disagreement was resolved by discussion with a fourth author (JL).

Two authors (JZ and CPW) evaluated the risk of bias in each study. The Methodological Quality and Synthesis of Case Series and Case Reports framework (16) was specifically employed for case series to evaluate bias in selection, ascertainment, causality, and reporting domains. Assessment was based on five binary criteria questions: (1) Did the patients in the study represent the full range of cases seen at the medical center? (2) Was the exposure adequately identified? (3) Was the outcome accurately determined? (4) Were other potential diagnoses ruled out? (5) Was all important information cited in the report? Each question was scored, with 5 indicating high quality, 4 moderate quality, and a score less than 3 indicative of low quality. Any disagreement was resolved by discussion with a third author (CXH).

### 2.4 Statistical analysis

Data were aggregated and presented using descriptive statistics. For normally distributed continuous variables, the mean ± standard deviation is presented. Non-normally distributed continuous variables are summarized using the median (interquartile range). Categorical variables are expressed as counts (percentages). Spearman’s rank correlation coefficient was used to assess the relationship between time to re-epithelization and other variables. For comparisons between two groups, the Student’s t-test was used for normally distributed continuous variables, while the Mann-Whitney U test was applied for non-normally distributed continuous variables. Fisher’s exact test was employed for categorical variables in two-group comparisons. For comparisons among three or more groups, one-way ANOVA was used for normally distributed continuous variables, the Kruskal-Wallis test for non-normally distributed continuous variables, and the Fisher-Freeman-Halton exact test for categorical variables. When overall differences were significant, *post-hoc* pairwise comparisons were conducted using Bonferroni correction to adjust for multiple comparisons. All statistical analyses were performed using SPSS version 27.0 (IBM Corp., Armonk, NY, USA). A *p*-value < 0.05 was considered statistically significant for all tests, except where adjusted for multiple comparisons as noted. All statistical tests were two-sided. Specific statistical methods for each analysis are indicated in the respective tables and figures.

## 3 Results

**Figure 1:**
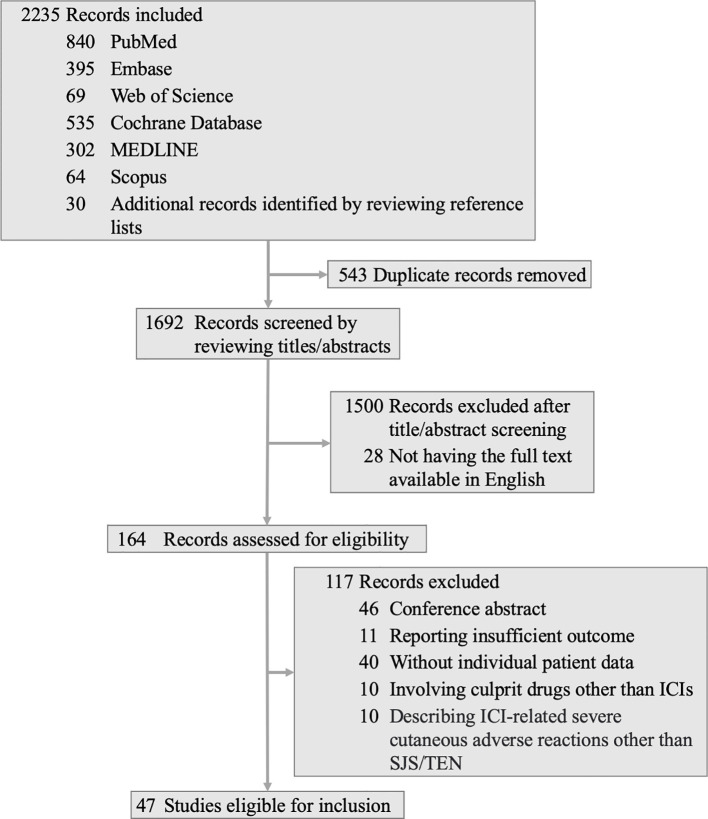
Flow diagram for search strategy and screening procedure. ICI, immune checkpoint inhibitor; SJS, Stevens-Johnson syndrome; TEN, toxic epidermal necrolysis.

Our comprehensive search identified 2,205 articles, augmented by 30 additional records from reference lists. We excluded 543 duplicates, 1,500 records based on title and abstract screenings, and 28 that were not available in English. From the 164 articles assessed in full text, 117 were excluded for reasons including the presence of 46 conference abstracts, 11 articles with inadequate outcome data, 40 lacking individual patient data, 10 involving culprit drugs other than ICIs, and 10 describing ICI-related severe cutaneous adverse reactions other than SJS/TEN. Ultimately, 47 articles (8, 17–62) encompassing data from 50 patients met our inclusion criteria. The details of the selection procedure are presented in **Figure 1**.

### 3.1 Study and patient characteristics

We compiled comprehensive data on study and patient characteristics in **Table 1** and **Supplementary Table 2**. The cohort included 18 patients (36%) with SJS, 28 (56%) with TEN, and 4 (8%) with SJS/TEN overlap syndrome. The mean age was 63 ± 14 years, with a slight male predominance (27 [54%]). The most common underlying tumors were non-small cell lung cancer (17 [34%]), melanoma (8 [16%]), and gastrointestinal cancer (5 [10%]). Most patients had stage IV disease (37 [74%]). PD-L1 expression was reported in 8 cases, with a median value of 30% (1%-80%).

**Table 1:** Demographics and clinical characteristics of the patients with ICI-related SJS/TEN.

Characteristics	All patients (n = 50)
Age, years	63 ± 14
Sex, male	27 (54%)
Malignancy types	
Non-small cell lung cancer	17 (34%)
Melanoma	8 (16%)
Gastrointestinal cancer	5 (10%)
Urothelial carcinoma	4 (8%)
Hepatocellular carcinoma	4 (8%)
Renal cell carcinoma	3 (6%)
Nasopharyngeal carcinoma	2 (4%)
Thymoma	2 (4%)
Others^a^	5 (10%)
Cancer staging (n = 44)	
Stage IV	37 (74%)
Stage II or III	7 (14%)
PD-L1 expression (n = 8)	30% (1%-80%)
ICI regimens	
Anti-PD-1	41 (82%)
Anti-PD-1 and anti-CTLA-4^b^	6 (12%)
Anti-PD-L1	2 (4%)
Anti-CTLA-4	1 (2%)
Suspected culprit ICIs	
Pembrolizumab	20 (40%)
Nivolumab	12 (24%)
Sintilimab	8 (16%)
Nivolumab combined with ipilimumab	5 (10%)
Atezolizumab	2 (4%)
Others^c^	3 (6%)
Concurrent treatments within 4 weeks prior to onset (n = 22)	
Chemotherapy	12 (24%)
Platinum	9 (18%)
Taxanes	5 (10%)
Radiation therapy	6 (12%)
Antibiotics^d^	5 (10%)
Targeted agents^e^	4 (8%)
Accompanying other irAEs (n = 8)	
Hepatitis	3 (6%)
Myocarditis and/or myositis	3 (6%)
Esophagogastroenteritis	2 (4%)
Others^f^	3 (6%)

Data are presented as mean ± standard deviation, median (interquartile range), or counts (percentages) as appropriate.

a Other malignancy types include one case each of cervical squamous cell carcinoma, intrahepatic cholangiocarcinoma, lymphoma, tongue cancer, and squamous cell vulvar carcinoma.

b Includes a PD-1/CTLA-4 bi-specific antibody, cadonilimab.

c Includes one case each of cadonilimab, ipilimumab, and toripalimab.

d Includes one case each of piperacillin/tazobactam, sulfamethoxazole/trimethoprim, sulfamethoxazole/trimethoprim with allopurinol, an anti-tuberculosis regimen of isoniazid, rifampicin, ethambutol, and pyrazinamide, and an unspecified antibiotic.

e Includes lenvatinib (n = 3) and osimertinib (n = 1).

f Includes one case each of pneumonia, hypophysitis, and thyroiditis.

CTLA-4, cytotoxic T lymphocyte-associated antigen 4; ICIs, immune checkpoint inhibitors; irAEs, immune-related adverse events; PD-1, programmed cell death protein-1; PD-L1, programmed cell death-ligand 1.

ICI regimens were primarily anti-PD-1 (41 [82%]), followed by combination therapies of anti-PD-1 and anti-CTLA-4 (6 [12%]), anti-PD-L1 (2 [4%]), and anti-CTLA-4 (1 [2%]). The most frequently administered ICIs were pembrolizumab (20 [40%]), nivolumab (12 [24%]), and sintilimab (8 [16%]).

Concurrent medications within 4 weeks before SJS/TEN onset were reported in 22 cases (44%), including chemotherapy, radiotherapy, antibiotics, and targeted agents. Concomitant irAEs were noted in 8 patients (16%), presenting as hepatitis, myocarditis and/or myositis, and gastroenteritis.

### 3.2 Clinical manifestations

The clinical characteristics of patients with ICI-related SJS/TEN are provided in **Table 2** and **Supplementary Table 3**. SJS/TEN typically occurred after a median of 2 cycles (1-3) of ICI therapy, corresponding to a median latency period of 23 days (14-61) from ICI initiation to symptom onset. Preceding SJS/TEN, 6 patients (12%) developed grade 1-2 maculopapular rashes or lichenoid dermatitis, which partially or completely resolved with topical or systemic corticosteroids. Despite this resolution, the patients developed SJS/TEN upon continued ICI therapy.

**Table 2:** Clinical manifestations of the patients with ICI-related SJS/TEN.

Characteristics	All patients (n = 50)
Time from ICI initiation to SJS/TEN onset, days	23 (14-61)
Cycles from ICI initiation to SJS/TEN onset	2 (1-3)
Preceding maculopapular rash or lichenoid dermatitis	6 (12%)
Clinical presentations	
Subtypes	
SJS	18 (36%)
TEN	28 (56%)
SJS/TEN overlap syndrome	4 (8%)
Epidermal detachment area, % of BSA (n = 31)	30% (15%-80%)
Fever (n = 21)	
Yes	17 (34%)
No	4 (8%)
Mucosal involvement	42 (84%)
Oral	39 (78%)
Ocular	25 (50%)
Genital	15 (30%)
Histopathologic findings consistent with SJS/TEN	40 (80%)
SCORTEN (0-7) (n = 23)	3 (3-4)
Predicted mortality rate based on SCORTEN	32% (32%-62%)

Data are presented as median (interquartile range), or counts (percentages) as appropriate.

BSA, body surface area; ICI, immune checkpoint inhibitor; SCORTEN, severity-of-illness score for toxic epidermal necrolysis; SJS, Stevens-Johnson syndrome; TEN, toxic epidermal necrolysis.

The median epidermal detachment area peaked at 30% of BSA (15%-80%). Fever accompanied 17 cases (34%), with mucosal involvement observed in 42 cases (84%); oral mucosa was implicated in 39 cases (78%), ocular in 25 cases (50%), and genital in 15 cases (30%). Histopathological confirmation of SJS/TEN was reported in 40 cases (80%). SCORTEN were available for 23 cases (46%), with a median score of 3 (3-4), correlating with a predicted mortality rate of 32% (32%-62%).

### 3.3 Management and outcomes

**Table 3** and **Supplementary Table 4** provide a comprehensive overview of management and outcomes for patients with ICI-related SJS/TEN. Systemic corticosteroids were the mainstay of treatment, administered in 46 cases (92%) at a median prednisone equivalent dose of 1.9 (1.0-2.5) mg/kg/day for about 30 (25-53) days. IVIG was administered in 25 cases (50%), with a median cumulative dose of 2.0 (2.0-2.9) g/kg. Cyclosporine was used in 9 cases (18%), with an average dose of 3.9 ± 1.1 mg/kg/day and an average duration of 16 ± 6 days. Tumor necrosis factor-α (TNF-α) inhibitors were used in 6 cases (12%), including infliximab, adalimumab, and etanercept. Other treatments included methylprednisolone 0.5-1 g pulse therapy, plasma exchange, mycophenolate mofetil, and granulocyte colony-stimulating factor.

**Table 3:** Management and outcomes of the patients with ICI-related SJS/TEN.

Characteristics	All patients (n = 50)
Systemic corticosteroids	
Yes	46 (92%)
Prednisone equivalent dose, mg/kg/day (n = 23)	1.9 (1.0-2.5)
Corticosteroid duration, days (n = 27)	30 (25-53)
Second-line treatments	
Intravenous immunoglobulins	25 (50%)
Cumulative dose, g/kg (n = 16)	2.0 (2.0-2.9)
Cyclosporine	9 (18%)
Dose, mg/kg/day (n = 9)	3.9 ± 1.1
Duration, days (n = 6)	16 ± 6
Tumor necrosis factor-α inhibitors	6 (12%)
Infliximab, 5 mg/kg intravenously once	2 (4%)
Adalimumab, 80 mg once or 40 mg twice subcutaneously	3 (6%)
Etanercept, 25 mg subcutaneously twice a week	1 (2%)
Other treatments	
Methylprednisolone pulse therapy 0.5-1 g for 3 days	2 (4%)
Plasma exchange	2 (4%)
Mycophenolate mofetil	2 (4%)
Granulocyte colony-stimulating factor	1 (2%)
SJS/TEN outcomes	
Recovered	40 (80%)
Time from onset to re-epithelization, days (n = 23)	30 (24-34)
Deceased	10 (20%)
Time from onset to death, days (n = 9)	28 (11-38)
Causes of death (n = 8)	
Infections	3 (30%)
Tumor progression	3 (30%)
Heart failure	1 (10%)
Hypovolemic shock	1 (10%)
Objective tumor response (n = 27)	
Progressive disease	15 (30%)
Stable disease	8 (16%)
Partial response	3 (6%)
Complete response	1 (2%)
Duration from SJS/TEN onset to death in recovered patients, days (n = 7)	95 ± 36
Causes of death	
Tumor progression	4 (57%)
Infections	2 (29%)
Pneumonia	1 (14%)

Data are presented as mean ± standard deviation, median (interquartile range), or counts (percentages) as appropriate.

ICI, immune checkpoint inhibitor; SJS, Stevens-Johnson syndrome; TEN, toxic epidermal necrolysis.

Of the 50 patients reviewed, 40 (80%) recovered, while 10 (20%) succumbed to SJS/TEN, with the mortality rate significantly higher in TEN than SJS or overlap syndrome (32% vs. 5%, *p* = 0.029). The median time from SJS/TEN onset to death was 28 (11-38) days. Infections and tumor progression were the most common causes of death.

**Figure 2:**
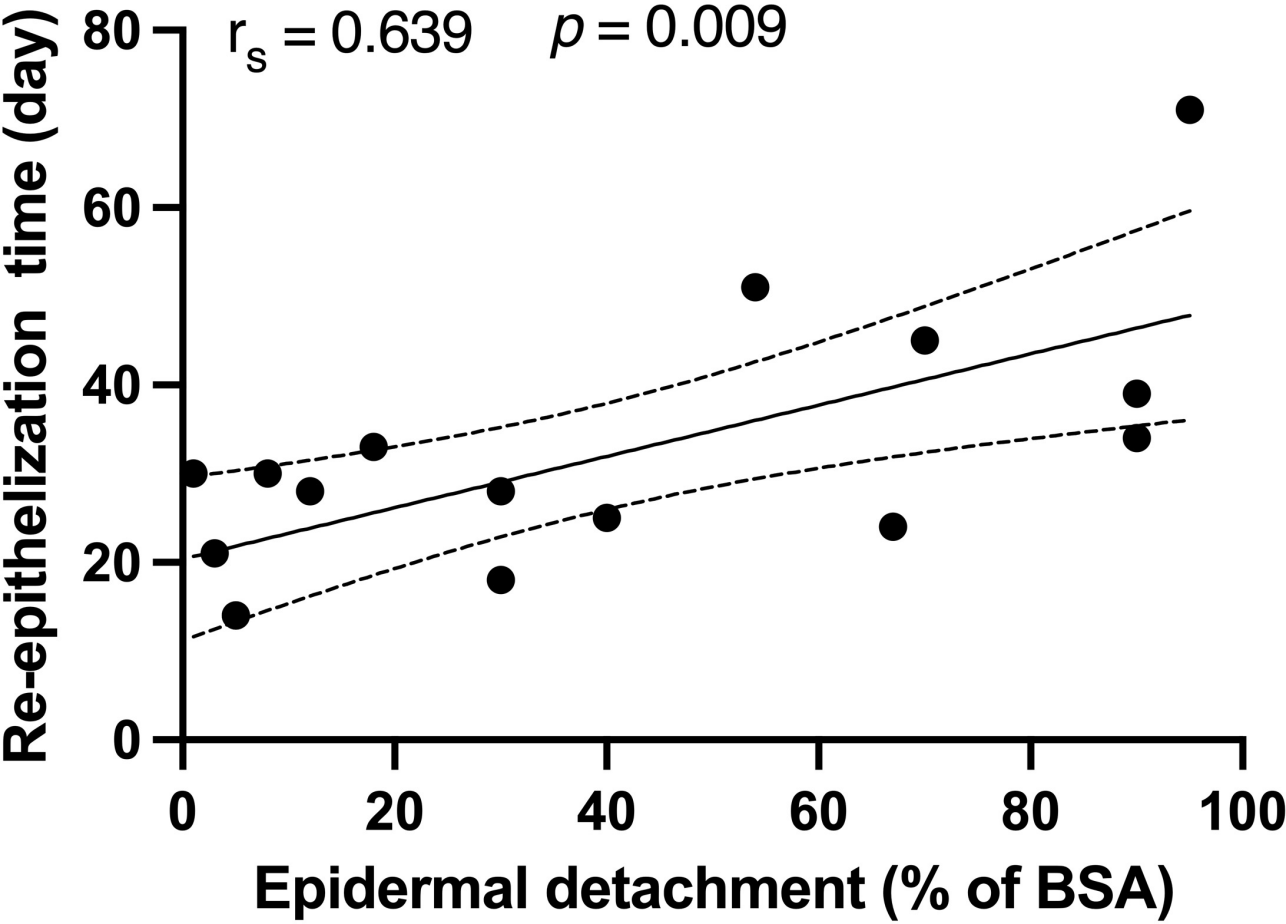
Correlations between the re-epithelization time and epidermal detachment area in recovered patients. Spearman’s rank correlation coefficient was used to assess the relationship between re-epithelization time and epidermal detachment area. BSA, body surface area.

The median duration from SJS/TEN onset to re-epithelization for survivors was 30 (24-34) days. A positive correlation was found between the re-epithelization time and the epidermal detachment area (r_s_ = 0.639, *p* = 0.009), as depicted in **Figure 2**. Notably, no significant correlations were found between re-epithelization time and patient demographics, SCORTEN, or treatment modalities. Among the survivors of SJS/TEN, subsequent deaths within a mean duration of 95 days were attributed primarily to tumor progression and infections.

Objective tumor response to ICIs was assessed in 27 patients, with only 4 (8%) showing a partial or complete response, while the rest experienced disease progression (15 [30%]) or stability (8 [16%]).

### 3.4 Comparisons among different subgroups

#### 3.4.1 Comparison of clinical characteristics and treatment regimens between deceased and survived patients

Deceased patients exhibited a significant higher proportion of TEN (90% vs. 48%, *p* = 0.029) and a larger area of epidermal detachment (90% vs. 30% of BSA, *p* = 0.005) compared to survivors. Other clinical characteristics and treatment regimens did not differ significantly between the two groups. For further details, please refer to **Table 4**.

**Table 4:** Comparison of clinical characteristics and treatment regimens between deceased and survived patients with ICI-related SJS/TEN.

	Deceased (n = 10)	Survived (n = 40)	*p* value
Sex, male	6 (60%)	21 (53%)	0.736
Age, years	63 ± 14	63 ± 15	0.988
Patients with TEN	9 (90%)	19 (48%)	0.029
Epidermal detachment, % of BSA	90 (80-90)	30 (11-69)	0.005
PD-L1 expression, %	10 (6-45)	50 (1-85)	0.786
Patients with stage IV	6 (86%)	31 (84%)	1.000
Dual ICI therapy	2 (20%)	4 (10%)	0.586
Cycles from ICI start to onset	2 (1-2)	2 (1-3)	0.285
Latency period, days	22 (9-32)	23 (14-65)	0.369
Mucosal involvement	9 (90%)	33 (89%)	1.000
SCORTEN (range 0-7)	3 (3-5)	3 (3-4)	0.870
Treatment regimens			
Corticosteroids combined with other treatments	8 (80%)	21 (53%)	0.136
Corticosteroid monotherapy	1 (10%)	16 (40%)	
Non-corticosteroid therapies	1 (10%)	3 (8%)	
Prednisone equivalent dose, mg/kg/day	1.0 (1.0-2.6)	1.9 (1.2-2.5)	0.491
Corticosteroid duration, days	39 (n = 1)	30 (24-54)	0.667

Data are presented as mean ± standard deviation, median (interquartile range), or number (percentage) as appropriate. *P* values were calculated using two-tailed Student’s t-test for normally distributed continuous variables, Mann-Whitney U test for non-normally distributed continuous variables, and Fisher’s exact test or Fisher-Freeman-Halton exact test for categorical variables. BSA, body surface area; ICI, immune checkpoint inhibitor; PD-L1, programmed death-ligand 1; SCORTEN, severity-of-illness score for toxic epidermal necrolysis; SJS, Stevens-Johnson syndrome; TEN, toxic epidermal necrolysis.

#### 3.4.2 Comparison of clinical characteristics and outcomes among different therapeutic regimens

We divided the treatment regimens into three groups: corticosteroids combined with other therapies (including IVIG, cyclosporine, TNF-α inhibitors, etc.), corticosteroid monotherapy, and non-corticosteroid therapies. A significant difference was observed in the proportion of TEN patients among the three groups (*p* = 0.010). Notably, the combination therapy group had a significantly higher proportion of TEN patients compared to the corticosteroid monotherapy group (72% vs. 29%, *p* = 0.005). The combination therapy group also showed a larger epidermal detachment area (69%) compared to corticosteroid monotherapy (20%) and non-corticosteroid therapy (30%) groups, although this difference was not statistically significant. The mortality rates were 28%, 6%, and 25% in the combination therapy, corticosteroid monotherapy, and non-corticosteroid therapy groups, respectively (*p* = 0.136). The median re-epithelization time was 30, 30, and 25 days in the three groups, respectively (*p* = 0.537). See **Table 5** for details.

**Table 5:** Comparison of clinical characteristics and outcomes among patients with ICI-related SJS/TEN receiving different therapeutic regimens.

	Corticosteroid combination therapy (n = 29)	Corticosteroid monotherapy (n = 17)	Non-corticosteroid therapies (n = 4)	*p* value
Sex, male	15 (52%)	9 (53%)	3 (75%)	0.755
Age, years	62 ± 16	65 ± 10	58 ± 7	0.561
Patients with TEN	21 (72%)	5 (29%)	2 (50%)	0.010
Epidermal detachment, % of BSA	69 (30-90)	20 (9-61)	30 (17-55)	0.146
Patients with stage IV	23 (92%)	10 (67%)	4 (100%)	0.112
Dual ICI therapy	3 (10%)	2 (12%)	1 (25%)	0.513
Cycles from ICI start to onset	2 (1-3)	2 (1-3)	2 (2-3)	0.619
Latency period, days	23 (14-60)	17 (9-58)	25 (23-64)	0.371
SCORTEN (range 0-7)	3 (3-5)	3 (3-3)	4 (3-5)	0.623
Mortality	8 (28%)	1 (6%)	1 (25%)	0.136
Re-epithelization time, days	30 (23-35)	30 (28-51)	25 (21-28)	0.537

Data are presented as mean ± standard deviation, median (interquartile range), or number (percentage) as appropriate. *P* values were calculated using one-way ANOVA for normally distributed continuous variables, Kruskal-Wallis test for non-normally distributed continuous variables, and Fisher-Freeman-Halton exact test for categorical variables. Pairwise comparisons using Bonferroni correction (adjusted α = 0.05/3 = 0.017) showed that the corticosteroid combination therapy group had a significantly higher proportion of TEN patients compared to the corticosteroid monotherapy group (*p* = 0.005). No significant differences were found between corticosteroid monotherapy and non-corticosteroid therapy groups (*p* = 0.574) or between corticosteroid combination therapy and non-corticosteroid therapy groups (*p* = 0.567). BSA, body surface area; ICI, immune checkpoint inhibitor; SCORTEN, severity-of-illness score for toxic epidermal necrolysis; SJS, Stevens-Johnson syndrome; TEN, toxic epidermal necrolysis.

#### 3.4.3 Comparison of clinical characteristics and outcomes among different ICI therapies

All patients receiving dual ICI therapy developed TEN, a significantly higher incidence (100%) compared to those receiving single ICI therapy (50%, *p* = 0.028). The dual ICI group also exhibited a more extensive epidermal detachment area (74% vs. 30% of BSA, *p* = 0.105) and a higher mortality rate (33% vs. 18%, *p* = 0.586) compared to single ICI group, though these differences were not statistically significant. Re-epithelization time was similar between groups (34 vs. 30 days, *p* = 0.456). Details are presented in **Table 6**.

**Table 6:** Comparison of clinical characteristics and outcomes between patients with ICI-related SJS/TEN receiving dual and single ICI therapy.

	Dual ICI therapy (n = 6)	Single ICI therapy (n = 44)	*p* value
Sex, male	5 (83%)	22 (50%)	0.199
Age, years	53 ± 11	64 ± 14	0.077
Patients with TEN	6 (100%)	22 (50%)	0.028
Epidermal detachment, % of BSA	74 (48-90)	30 (11-80)	0.105
Cycles from ICI start to onset	2 (1-3)	2 (1-3)	0.819
Latency period, days	25 (6-77)	23 (14-60)	0.942
SCORTEN (range 0-7)	3 (3-5)	3 (3-4)	0.759
Mortality	2 (33%)	8 (18%)	0.586
Re-epithelization time, days	34 (25-48)	30 (21-33)	0.456

Data are presented as mean ± standard deviation, median (interquartile range), or number (percentage) as appropriate. *P* values were calculated using two-tailed Student’s t-test for normally distributed continuous variables, Mann-Whitney U test for non-normally distributed continuous variables, and Fisher’s exact test for categorical variables. BSA, body surface area; ICI, immune checkpoint inhibitor; SCORTEN, severity-of-illness score for toxic epidermal necrolysis; SJS, Stevens-Johnson syndrome; TEN, toxic epidermal necrolysis.

Among patients receiving single ICI therapies, those treated with sintilimab had a higher proportion of TEN (75% vs. 40-50%) and a larger epidermal detachment area (90% vs. 30-48% of BSA) compared to those treated with other agents, although these differences were not statistically significant. Mortality rates were comparable across all groups. Notably, re-epithelization time was significantly longer in sintilimab group (44 vs. 14-28 days in other groups, *p* = 0.036). However, *post-hoc* pairwise comparisons revealed no significant differences between any two groups. This discrepancy may be attributed to the limited sample size, resulting in reduced statistical power and potential instability of the results. Details are presented in **Table 7**.

**Table 7:** Comparison of clinical characteristics and outcomes among patients with ICI-related SJS/TEN receiving different single ICI therapies.

	Sintilimab (n = 8)	Pembrolizumab (n = 20)	Nivolumab (n =12)	Other single ICIs (n = 4)	*p* value
Sex, male	5 (63%)	11 (55%)	4 (33%)	2 (50%)	0.583
Age, years	67 ± 18	62 ± 13	66 ± 13	63 ± 16	0.754
Patients with TEN	6 (75%)	8 (40%)	6 (50%)	2 (50%)	0.417
Epidermal detachment, % of BSA	90 (80-93)	30 (7-60)	30 (14-60)	48 (10-78)	0.172
Cycles from ICI start to onset	2 (1-4)	1 (1-2)	2 (2-3)	3 (2-7)	0.128
Latency period, days	20 (12-68)	19 (8-62)	28 (22-53)	41 (19-161)	0.340
SCORTEN (range 0-7)	4 (3-7)	3 (3-3)	3 (3-4)	missing	0.368
Mortality	1 (13%)	3 (15%)	4 (33%)	0	0.539
Re-epithelization time, days	44 (33-65)	28 (20-31)	28 (21-30)	14 (n = 1)	0.036

Data are presented as mean ± standard deviation, median (interquartile range), or number (percentage) as appropriate. *P* values were calculated using one-way ANOVA for normally distributed continuous variables, Kruskal-Wallis test for non-normally distributed continuous variables, and Fisher-Freeman-Halton exact test for categorical variables. Post-hoc pairwise comparisons using Bonferroni correction revealed no significant differences between any two groups in re-epithelization time (all adjusted *p*-values > 0.05). Specifically, the adjusted *p*-values for pairwise comparisons were as follows: others vs. sintilimab (*p* = 0.096), pembrolizumab vs. sintilimab (*p* = 0.132), nivolumab vs. sintilimab (*p* = 0.218), others vs. nivolumab (*p* = 1.000), others vs. pembrolizumab (*p* = 1.000), and nivolumab vs. pembrolizumab (*p* = 1.000). BSA, body surface area; ICI, immune checkpoint inhibitor; SCORTEN, severity-of-illness score for toxic epidermal necrolysis; SJS, Stevens-Johnson syndrome; TEN, toxic epidermal necrolysis.

#### 3.4.4 Comparison of clinical characteristics and outcomes between stage IV and stage II/III patients

Stage IV patients tended to have a higher proportion of TEN (60% vs. 29%), a larger epidermal detachment area (35% vs. 21% of BSA), and a shorter latency period (23 vs. 60 days) compared to patients with stage II or III, though these differences were not statistically significant. Both groups showed similar mortality rates (16% vs. 14%) and re-epithelization time (30 vs. 28 days). Details are presented in **Table 8**.

**Table 8:** Comparison of clinical characteristics and outcomes between stage IV and stage II/III patients with ICI-related SJS/TEN.

	Stage IV (n = 37)	Stage II or III (n = 7)	*p* value
Sex, male	17 (46%)	4 (57%)	0.693
Age, years	61 ± 14	62 ± 18	0.927
Patients with TEN	22 (60%)	2 (29%)	0.217
Epidermal detachment, % of BSA	35 (17-80)	21 (11-63)	0.758
Cycles from ICI start to onset	2 (1-3)	3 (1-4)	0.243
Latency period, days	23 (14-62)	60 (10-70)	0.412
SCORTEN (range 0-7)	3 (3-5)	3 (3-3)	0.549
Mortality	6 (16%)	1 (14%)	1.000
Re-epithelization time, days	30 (20-35)	28 (28-50)	0.600

Data are presented as mean ± standard deviation, median (interquartile range), or number (percentage) as appropriate. *P* values were calculated using two-tailed Student’s t-test for normally distributed continuous variables, Mann-Whitney U test for non-normally distributed continuous variables, and Fisher’s exact test for categorical variables. BSA, body surface area; ICI, immune checkpoint inhibitor; SCORTEN, severity-of-illness score for toxic epidermal necrolysis; SJS, Stevens-Johnson syndrome; TEN, toxic epidermal necrolysis.

## 4 Discussion

Our systematic review provides a comprehensive examination of the clinical profiles, treatment strategies, and outcomes for patients with ICI-related SJS/TEN. This cohort, characterized by a mean age of 63 years and a slight male predominance (54%), primarily comprised patients with melanoma and non-small cell lung cancer. SJS/TEN typically manifested early in the treatment course, with a median onset of 23 days post-ICI initiation, usually after 1-2 cycles. The mainstay of treatment involved systemic corticosteroids and IVIG. Despite these interventions, the overall mortality rate was 20%, with TEN cases showing a notably higher rate of 32%. Infections and tumor progression emerged as the primary causes of death, with a median time from SJS/TEN onset to death of 28 days. Survivors demonstrated a median re-epithelization time of 30 days, positively correlated with the extent of epidermal detachment. Notably, deceased patients exhibited a significantly higher proportion of TEN and a larger epidermal detachment area compared to survivors. There were no significant differences in mortality or re-epithelization time among different treatment groups. Dual ICI therapy resulted in a higher TEN rate compared to single ICI therapy. Among single ICI therapies, patients treated with sintilimab showed trends towards a higher TEN rate, larger detachment area, and longer re-epithelization time compared to other groups. However, mortality rates were comparable across different ICI regimens.

The clinical manifestations of ICI-related SJS/TEN in our study largely align with those triggered by traditional drugs, showing no significant gender difference in incidence, a relatively short latency period, and frequent occurrences of fever and mucositis (63, 64). The median latency period for ICI-related cases in our study was 23 days, falling within the classic 4-28 day range for SJS/TEN (63, 64). While mucosal involvement patterns in ICI-related SJS/TEN were similar to classic cases, the incidence was lower: oral mucosa was implicated in 78% of cases (vs. 90% in classic SJS/TEN), ocular in 50% (vs. 84%), and genital in 30% (vs. 60-70%) (65–68). This lower incidence might be due to underreporting or could indicate a distinctive feature of ICI-related cases. Notably, some patients with extensive epidermal detachment did not exhibit mucosal involvement, suggesting unique characteristics of ICI-related SJS/TEN (22, 36). A subset of patients initially developed mild maculopapular rashes or lichenoid dermatitis early in their ICI treatment. Despite initial improvement with corticosteroids, these conditions progressed to SJS/TEN with continued therapy (25, 47). This progression, uncommon in traditional drug-induced cases, underscores the need for heightened vigilance in monitoring patients exhibiting such symptoms after ICI therapy.

Our study identified that about half of the patients with ICI-related SJS/TEN had received other medications within the month before the onset, including antibiotics and targeted drugs. These agents, such as allopurinol (69), sulfamethoxazole/trimethoprim (70), lenvatinib (24), and osimertinib (71), are well known to precipitate SJS/TEN. When SJS/TEN develops alongside ICI therapy and these medications, pinpointing the primary culprit drug becomes a substantial challenge. Sole reliance on clinical expertise or the ALDEN score might not yield a definitive determination of drug causality, which is pivotal in deciding the continuity of ICI treatment. Diagnostic approaches to ascertain the culprit drug in SJS/TEN encompass both *in vivo* and *in vitro* tests. *In vivo* assessments, like delayed intradermal tests or drug provocation tests, are generally contraindicated due to the life-threatening nature of SJS/TEN (72–74). *In vitro* assays such as lymphocyte transformation tests and enzyme-linked immunospot assays have exhibited high specificity and modest sensitivity in attributing causality to traditional drugs in the context of severe cutaneous adverse reactions (75–77). Nevertheless, their application for establishing a link between ICIs and SJS/TEN has not been documented. Additionally, the association between specific human leukocyte antigen (HLA) alleles and conventional drug-induced SJS/TEN is well-established, with strong correlations identified—such as HLA-B*58:01 with allopurinol-induced cases (78) and HLA-B*15:02 with carbamazepine-induced cases (79, 80). However, the potential link of certain HLA alleles with ICI-related SJS/TEN is yet to be elucidated.

In the diagnosis of ICI-related SJS/TEN, it is imperative to rule out other blistering dermatoses, including bullous lichenoid dermatitis, bullous radiation recall dermatitis, bullous pemphigoid, and paraneoplastic pemphigus. Each condition presents with distinct clinical manifestations, pathological findings, and carries a different prognosis, as outlined in **Table 9** (81, 82). In cases presenting with atypical clinical features, an in-depth histopathological examination, supplemented by immunofluorescence studies and circulating autoantibody assays, is crucial to differentiate these conditions accurately. ICI-induced SJS/TEN requires permanent discontinuation of the culprit drug. In contrast, for other bullous dermatoses, the potential resumption of ICI therapy may be considered under careful evaluation (10).

**Table 9:** Key points for the differential diagnosis of ICI-related SJS/TEN versus other blistering dermatoses.

	SJS/TEN	Bullous lichenoid dermatitis	Bullous radiation recall dermatitis	Bullous pemphigoid	Paraneoplastic pemphigus
Median days from ICI initiation to onset	23 (IQR 14-61)	70 (IQR 23-251)	Not reported	365 (range 60-810)	Not reported
Onset	Acute	Gradual	Acute	Gradual	Gradual
Fever	Often high fever	Typically no fever	Typically no fever	Typically no fever	Typically no fever
Rash features	Erythemas, bullae, and skin detachment	Scaly papules and plaques alongside blisters	Erythemas and blisters, aggravated at the site of radiation	Erythemas, blisters and bullae	Erythemas, bullae, and skin detachment
Nikolsky sign	Positive	Negative	Negative	Negative	Positive
Mucosal involvement	Severe	Mild to severe	Mild or absent	Mild or absent	Severe
Histopathologic features	Full thickness epidermal necrosis with sparse dermal inflammation	Interface dermatitis with band-like dermal lymphocyte infiltration	Interface dermatitis with perivascular lymphocyte infiltration	Subepidermal cleft with eosinophils within the blister cavity and dermis	Intraepidermal bullae or subepidermal bullae with acantholysis, epidermal necrosis and liquefaction degeneration
Direct immunofluorescence findings	Negative	Negative	Negative	Linear C3 and/or IgG along the basement membrane zone	Deposition of IgG and/or C3 along the keratinocyte cell surfaces (intercellular) and along the basement membrane zone (linear)
Serum autoantibodies	Negative	Negative	Negative	Positive anti-BP180 and/or BP230	Multiple autoantibodies against desmoglein 1, desmoglein 3, and BP180 etc.

ICI, immune checkpoint inhibitor; IQR, interquartile range; SJS, Stevens-Johnson syndrome; TEN, toxic epidermal necrolysis.

While the role of corticosteroids in SJS/TEN treatment is debated, current guidelines still advocate for their use as the cornerstone of therapy in ICI-related cases, with methylprednisolone dosages suggested at 1-2 mg/kg/day (9, 10). For non-responders, adjunctive therapies such as IVIG, cyclosporine, or TNF-α inhibitors are considered (9, 10). Although the preference for second-line agents is not yet standardized, cyclosporine and TNF-α inhibitors are showing promising results (83–86). Considering the rapid progression of SJS/TEN—where peak skin detachment typically occurs within a median of 8 days—and the prognostic significance of the extent of skin loss, swift initiation of corticosteroids is crucial (87). This initial treatment may be effectively supplemented with TNF-α inhibitors or cyclosporine during the acute phase to halt the inflammatory cascade, limit epidermal necrosis, and expedite recovery. However, a prolonged course of corticosteroids may impede wound repair and elevate infection risks (88). It is therefore recommended to begin tapering corticosteroids as signs of recovery appear, such as the cessation of new erythema or blistering and the start of re-epithelization.

Our study found no significant differences in mortality rates or re-epithelization time among groups treated with combination corticosteroid therapy, corticosteroid monotherapy, or non-corticosteroid therapies. However, the combination therapy group showed a higher proportion of TEN cases and larger epidermal detachment area compared to the other groups. This observation likely reflects the tendency to employ more aggressive treatment strategies for patients with severe disease presentations. These findings underscore the urgent need for prospective, well-designed clinical trials to investigate the efficacy, safety, and optimal timing of these therapeutic approaches in managing ICI-related SJS/TEN.

The acute mortality rate for ICI-related SJS/TEN observed in our study was 20%, notably lower than both the SCORTEN-predicted mortality rate of 32% and reported mortality rates for cancer patients with SJS/TEN (89). This decrease in mortality may be attributed to enhanced recognition and prompt intervention by oncologists and dermatologists, coupled with advancements in supportive care (90). However, the mortality rate for TEN specifically persists at 32%, revealing resistance to current therapies among certain patients and highlighting the urgent need for more efficacious treatment approaches to enhance survival and prognosis. Our study found that the median time from onset to death for patients with ICI-related SJS/TEN was 28 days. Some surviving patients died from tumor progression or infection, on average, 95 days after onset. This timeline is consistent with that observed in classic SJS/TEN patients. In the RegiSCAR cohort study, Sekula et al. reported that most deaths in SJS/TEN occurred within 6 weeks, with mortality rates continuing to rise over the first year (91).

The predominant causes of death in our cohort were infections and tumor progression. Reported mortality predictors in SJS/TEN included factors such as advanced age, existing comorbidities, hematologic cancers, septicemia, pneumonia, and renal impairment (92). Sepsis has been pinpointed as the leading cause of death in these patients, with TEN, diabetes, and intensive care unit admission identified as key risk factors (93–95). Our study provides additional insights into prognostic factors specific to ICI-related cases. We found that deceased patients exhibited a significantly higher rate of TEN and larger epidermal detachment area compared to survivors. This finding suggests that the extent of epidermal detachment is a crucial determinant of prognosis in ICI-related cases, potentially due to the increased risk of infection and metabolic disturbances associated with extensive skin involvement. While SCORTEN remains the gold standard for prognostic evaluation in SJS/TEN (14), emerging research has identified potential biomarkers. Chemokine CCL27 and interleukin-15 are associated with disease severity and mortality (96, 97). Additionally, RIP3 expression may act as a diagnostic marker indicative of epidermal necrosis (98). These biomarkers, however, have not yet gained widespread clinical adoption and warrant further study.

Our data indicates that patients with ICI-related SJS/TEN typically experience re-epithelization within 30 days, a process closely linked to the degree of epidermal detachment. This duration is notably longer than the 12-17 days reported for classic SJS/TEN in the literature. For instance, Wang et al. reported re-epithelization times of 13.8 and 16.5 days for etanercept and corticosteroid groups, respectively, in a randomized controlled trial (83). Zhang et al., in a multicenter retrospective study, found times ranging from 12 to 13.5 days across different treatment groups (86). Krajewski et al.’s meta-regression analysis reported an average of 13 days (99). This discrepancy can be attributed to several factors. Firstly, our study defines re-epithelization time as the duration from SJS/TEN onset to complete skin healing, potentially encompassing a longer period compared to studies measuring from treatment initiation or using unclear definitions. Secondly, patients with ICI-related SJS/TEN are typically older, and often suffer from conditions such as anemia, hypoalbuminemia, or malnutrition, which may contribute to prolonged healing times.

Our study revealed that patients receiving dual ICI therapy experienced more severe SJS/TEN and showed a trend towards higher mortality rates compared to those on single ICI therapy. This finding aligns with broader observations in immunotherapy research, where multiple analyses have consistently shown that dual ICI therapy significantly increases the risk of grade ≥ 3 irAEs compared to single ICI therapy (100–102). Interestingly, our research also uncovered variations in adverse event profiles among different single ICI therapies. Notably, patients treated with sintilimab who developed SJS/TEN exhibited more severe disease manifestations and longer re-epithelization time compared to those on other ICIs, although mortality rates were not significantly elevated. While our findings focus on SJS/TEN severity, a phase II randomized controlled trial has reported a higher incidence of rash with sintilimab compared to pembrolizumab in advanced non-small cell lung cancer treatment (103). These findings suggest that even within the category of single ICI therapies, different agents may carry varying risks for severe cutaneous adverse reactions.

This systematic review has several limitations that merit attention. Firstly, the exclusion of non-English literature may have omitted relevant studies, potentially affecting the comprehensiveness of our analysis. Secondly, a substantial limitation is that almost all the included literature consists of case reports, which are subject to publication bias and may not represent the full spectrum of ICI-related SJS/TEN cases. Thirdly, critical data such as SCORTEN, detailed treatment regimens, and precise timing of death or recovery were inconsistently reported across case reports. These gaps in information could obscure a comprehensive understanding of the disease course and treatment efficacy. Lastly, the sample size, while considerable given the rarity of ICI-related SJS/TEN, remains relatively small, limiting the generalizability and statistical power of our findings. This constraint is particularly pronounced in our subgroup analyses, where some groups comprised only one or two cases, leading to potentially unstable statistical results and insufficient power to detect meaningful differences. Despite these limitations, our study provides valuable insights into the clinical characteristics, management, and outcomes of ICI-related SJS/TEN. However, the findings should be interpreted with caution, and future prospective studies with larger, more homogeneous cohorts are necessary to validate and expand upon these results.

## 5 Conclusions

Our systematic review illuminates the complex landscape of ICI-related SJS/TEN, highlighting its rarity yet serious nature within oncological settings. The findings underscore the importance of early recognition, accurate diagnosis, and multidisciplinary management to mitigate potentially fatal outcomes. Our analysis suggests potential differences in disease severity and outcomes based on treatment regimens and specific ICI agents, emphasizing the need for tailored monitoring approaches.

However, the study’s limitations, primarily the reliance on case reports and small sample sizes, necessitate cautious interpretation of these findings. These constraints underscore the critical need for prospective, well-designed clinical trials with larger cohorts and standardized reporting. Such studies are essential to validate our observations, elucidate underlying mechanisms, and enhance treatment protocols for ICI-related SJS/TEN.

In conclusion, while our study provides valuable insights into the current landscape of this severe adverse event, it also serves as a call to action for more robust research to advance our understanding and management of ICI-related SJS/TEN in cancer immunotherapy.

## Data availability statement

The original contributions presented in the study are included in the article/**Supplementary Material**. Further inquiries can be directed to the corresponding author.

## Author contributions

JZ: Data curation, Formal analysis, Investigation, Methodology, Project administration, Visualization, Writing – original draft, Writing – review & editing. CW: Data curation, Formal analysis, Investigation, Methodology, Project administration, Visualization, Writing – original draft, Writing – review & editing. JL: Conceptualization, Funding acquisition, Supervision, Validation, Writing – original draft, Writing – review & editing. HZ: Writing – original draft, Writing – review & editing. CH: Conceptualization, Data curation, Formal analysis, Funding acquisition, Investigation, Methodology, Project administration, Supervision, Validation, Visualization, Writing – original draft, Writing – review & editing.
