# Supplementary material for: Stevens-Johnson syndrome and toxic epidermal necrolysis associated with immune checkpoint inhibitors: a systematic review
Source: Front Immunol. 2024 Jul 12;15:1414136. doi: 10.3389/fimmu.2024.1414136 (PMC11272453; doi:10.3389/fimmu.2024.1414136)
Supplement: Supplementary file 1 [file DataSheet_1.docx]

***Supplementary Material***

**Stevens-Johnson Syndrome and Toxic Epidermal Necrolysis Associated with Immune Checkpoint Inhibitors: A Systematic Review**

**Jia Zhou^1†^, Chuan-Peng Wang^2†^, Jun Li^1^, Han-Lin Zhang^1^, Chun-Xia He^1*^**

^1^Department of Dermatology, State Key Laboratory of Complex Severe and Rare Diseases, Peking Union Medical College Hospital, Chinese Academy of Medical Sciences and Peking Union Medical College, National Clinical Research Center for Dermatologic and Immunologic Diseases, Beijing, China.

^2^Department of Nephrology, Peking Union Medical College Hospital, Chinese Academy of Medical Sciences and Peking Union Medical College, Beijing, China.

*** Correspondence:**

Chun-Xia He

[hcxpumch@foxmail.com](mailto:hcxpumch@foxmail.com)

**Supplementary Table 1** Database Search Strategy

| Cochrane:  ('severe cutaneous adverse reaction':ti,ab,kw) OR (('reaction*' OR 'adverse' OR 'complications' OR 'toxicity' OR 'toxicities'):ti,ab,kw AND ('severe' OR 'acute' OR 'life-threatening'):ti,ab,kw AND ('skin' OR 'cutaneous' OR 'dermatologic'):ti,ab,kw) OR (('Stevens-Johnson Syndrome' OR 'SJS' OR 'toxic epidermal necrolysis' OR 'drug-induced hypersensitivity syndrome' OR 'DIHS' OR 'drug reaction with eosinophilia and systemic symptoms' OR 'DRESS' OR 'acute generalized exanthematous pustulosis' OR 'AGEP' OR 'exfoliative dermatitis' OR 'immunobullous disorder'):ti,ab,kw) AND ('immune checkpoint inhibitor' OR 'immune checkpoint inhibitors' OR 'Immune checkpoint inhibition' OR 'immune checkpoint blockade*' OR 'immune checkpoint blocking' OR immunotherapy OR 'immune related' OR PD-1 OR 'programmed cell death protein 1' OR Pembrolizumab OR Keytruda OR Nivolumab OR Opdivo OR Cemiplimab OR Libtayo OR PD-L1 OR 'programmed death ligand 1' OR Atezolizumab OR Tecentriq OR Avelumab OR Bavencio OR Durvalumab OR Imfinzi OR 'CTLA 4' OR 'cytotoxic T lymphocyte associated protein 4' OR Ipilimumab OR Yervoy):ti,ab,kw |
| --- |
| Embase:  ('article'/it OR 'review'/it) AND (('case report'/de OR 'clinical article'/de OR 'cohort analysis'/de OR 'controlled clinical trial'/de OR 'controlled study'/de OR 'double blind procedure'/de OR 'human'/de OR 'human cell'/de OR 'human tissue'/de OR 'major clinical study'/de OR 'multicenter study'/de OR 'prospective study'/de OR 'randomized controlled trial'/de OR 'retrospective study'/de) AND (('immune checkpoint inhibitor':ti,ab OR 'immune checkpoint inhibitors':ti,ab OR 'immune checkpoint inhibition':ti,ab OR 'immune checkpoint blockade*':ti,ab OR 'immune checkpoint blocking':ti,ab OR immunotherapy:ti,ab OR 'immune related':ti,ab OR 'pd 1':ti,ab OR 'programmed cell death protein 1':ti,ab OR pembrolizumab:ti,ab OR keytruda:ti,ab OR nivolumab:ti,ab OR opdivo:ti,ab OR cemiplimab:ti,ab OR libtayo:ti,ab OR 'pd l1':ti,ab OR 'programmed death ligand 1':ti,ab OR atezolizumab:ti,ab OR tecentriq:ti,ab OR avelumab:ti,ab OR bavencio:ti,ab OR durvalumab:ti,ab OR imfinzi:ti,ab OR 'ctla 4':ti,ab OR 'cytotoxic t lymphocyte associated protein 4':ti,ab OR ipilimumab:ti,ab OR yervoy:ti,ab) AND (('severe cutaneous adverse reaction'/exp OR 'severe cutaneous adverse reaction') OR ('stevens-johnson syndrome':ti,ab OR 'toxic epidermal necrolysis':ti,ab OR 'drug-induced hypersensitivity syndrome':ti,ab OR 'dihs':ti,ab OR 'drug reaction with eosinophilia and systemic symptoms':ti,ab OR 'dress':ti,ab OR 'acute generalized exanthematous pustulosis':ti,ab OR 'agep':ti,ab OR 'exfoliative dermatitis':ti,ab OR 'immunobullous disorder':ti,ab)))) |
| MEDLINE:  1  (('severe' or 'acute' or 'life-threatening') and ('skin' or 'cutaneous' or 'dermatologic') and ('reaction*' or 'adverse' or 'complications' or 'toxicit*')).mp. [mp=title, book title, abstract, original title, name of substance word, subject heading word, floating sub-heading word, keyword heading word, organism supplementary concept word, protocol supplementary concept word, rare disease supplementary concept word, unique identifier, synonyms, population supplementary concept word, anatomy supplementary concept word]  2  limit 1 to (abstracts and structured abstracts)  3  ((('Stevens-Johnson Syndrome' or 'SJS' or 'toxic epidermal necrolysis' or 'drug-induced hypersensitivity syndrome' or 'DIHS' or 'drug reaction with eosinophilia) and systemic symptoms') or 'DRESS' or 'acute generalized exanthematous pustulosis' or 'AGEP' or 'exfoliative dermatitis' or 'immunobullous disorder').mp. [mp=title, book title, abstract, original title, name of substance word, subject heading word, floating sub-heading word, keyword heading word, organism supplementary concept word, protocol supplementary concept word, rare disease supplementary concept word, unique identifier, synonyms, population supplementary concept word, anatomy supplementary concept word]  4  limit 3 to (abstracts and structured abstracts)  5  ('immune checkpoint inhibitor' or 'immune checkpoint inhibitors' or 'Immune checkpoint inhibition' or 'immune checkpoint blockade*' or 'immune checkpoint blocking' or immunotherapy or 'immune related' or PD-1 or 'programmed cell death protein 1' or Pembrolizumab or Keytruda or Nivolumab or Opdivo or Cemiplimab or Libtayo or PD-L1 or 'programmed death ligand 1' or Atezolizumab or Tecentriq or Avelumab or Bavencio or Durvalumab or Imfinzi or 'CTLA 4' or 'cytotoxic T lymphocyte associated protein 4' or Ipilimumab or Yervoy).mp. [mp=title, book title, abstract, original title, name of substance word, subject heading word, floating sub-heading word, keyword heading word, organism supplementary concept word, protocol supplementary concept word, rare disease supplementary concept word, unique identifier, synonyms, population supplementary concept word, anatomy supplementary concept word]  6  limit 5 to (abstracts and structured abstracts)  7  2 or 4  8  6 and 7  9  limit 8 to (case reports or classical article or clinical study or clinical trial, all or comparative study or controlled clinical trial or dataset or meta analysis or multicenter study or observational study or randomized controlled trial or "review" or "systematic review") |
| PubMed:  ("immune checkpoint inhibitor*"[Title/Abstract] OR "Immune Checkpoint Inhibitors"[Pharmacological Action] OR "Immune Checkpoint Inhibitors"[MeSH Terms] OR "immune checkpoint inhibition"[Title/Abstract] OR "immune checkpoint blockade*"[Title/Abstract] OR "immune checkpoint blocking"[Title/Abstract] OR "Pembrolizumab"[Title/Abstract] OR "Pembrolizumab"[Supplementary Concept] OR "Keytruda"[Title/Abstract] OR "Nivolumab"[Title/Abstract] OR "Nivolumab"[MeSH Terms] OR "Opdivo"[Title/Abstract] OR "Cemiplimab"[Title/Abstract] OR "Cemiplimab"[Supplementary Concept] OR "Libtayo"[Title/Abstract] OR "PD-1"[Title/Abstract] OR "programmed cell death protein 1"[Title/Abstract] OR "Programmed Cell Death 1 Receptor"[MeSH Terms] OR "PD-L1"[Title/Abstract] OR "programmed death ligand 1"[Title/Abstract] OR "Atezolizumab"[Title/Abstract] OR "Atezolizumab"[Supplementary Concept] OR "Tecentriq"[Title/Abstract] OR "Avelumab"[Title/Abstract] OR "Avelumab"[Supplementary Concept] OR "Bavencio"[Title/Abstract] OR "Durvalumab"[Title/Abstract] OR "Durvalumab"[Supplementary Concept] OR "Imfinzi"[Title/Abstract] OR "ctla 4"[Title/Abstract] OR "CTLA-4 Antigen"[MeSH Terms] OR "cytotoxic t lymphocyte associated protein 4"[Title/Abstract] OR "Ipilimumab"[Title/Abstract] OR "Ipilimumab"[MeSH Terms] OR "Yervoy"[Title/Abstract] OR "immunotherapy"[Title/Abstract] OR "immune related"[Title/Abstract]) AND ("severe cutaneous adverse reaction*"[Title/Abstract] OR ((("severe"[Title/Abstract] OR "acute"[Title/Abstract] OR "life threatening"[Title/Abstract]) AND ("skin"[Title/Abstract] OR "cutaneous"[Title/Abstract] OR "dermatologic"[Title/Abstract]) AND ("reaction"[Title/Abstract] OR "adverse"[Title/Abstract] OR "complications"[Title/Abstract] OR "toxicity"[Title/Abstract] OR "toxicities"[Title/Abstract])) OR ((("stevens johnson syndrome"[Title/Abstract] OR "sjs"[Title/Abstract] OR "toxic epidermal necrolysis"[Title/Abstract] OR "drug induced hypersensitivity syndrome"[Title/Abstract] OR "dihs"[Title/Abstract] OR "drug reaction with eosinophilia"[Title/Abstract]) AND "systemic symptoms"[Title/Abstract]) OR "dress"[Title/Abstract] OR "acute generalized exanthematous pustulosis"[Title/Abstract] OR "agep"[Title/Abstract] OR "exfoliative dermatitis"[Title/Abstract] OR "immunobullous disorder"[Title/Abstract]))) |
| SCOPUS:  (TITLE-ABS-KEY ( 'severe AND cutaneous AND adverse AND reaction' ) OR TITLE-ABS-KEY ( ( 'severe' OR 'acute' OR 'life-threatening' ) AND ( 'skin' OR 'cutaneous' OR 'dermatologic' ) AND ( 'reaction*' OR 'adverse' OR 'complications' OR 'toxicity' OR 'toxicities' ) ) OR TITLE-ABS-KEY ( ( 'stevens-johnson AND syndrome' OR 'sjs' OR 'toxic AND epidermal AND necrolysis' OR 'drug-induced AND hypersensitivity AND syndrome' OR 'dihs' OR 'drug AND reaction AND with AND eosinophilia AND systemic AND symptoms' OR 'dress' OR 'acute AND generalized AND exanthematous AND pustulosis' OR 'agep' OR 'exfoliative AND dermatitis' OR 'immunobullous AND disorder' ) ) AND TITLE-ABS-KEY ( ( 'immune AND checkpoint AND inhibitor' OR 'immune AND checkpoint AND inhibitors' OR 'immune AND checkpoint AND inhibition' OR 'immune AND checkpoint AND blockade*' OR 'immune AND checkpoint AND blocking' OR immunotherapy OR 'immune AND related' OR pd-1 OR 'programmed AND cell AND death AND protein AND 1' OR pembrolizumab OR keytruda OR nivolumab OR opdivo OR cemiplimab OR libtayo OR pd-l1 OR 'programmed AND death AND ligand AND 1' OR atezolizumab OR tecentriq OR avelumab OR bavencio OR durvalumab OR imfinzi OR 'ctla AND 4' OR 'cytotoxic AND t AND lymphocyte AND associated AND protein AND 4' OR ipilimumab OR yervoy ) ) ) |
| WOS:  (((TI=(severe cutaneous adverse reaction)) OR TI=(('severe' OR 'acute' OR 'life-threatening') AND ('skin' OR 'cutaneous' OR 'dermatologic') AND ('reaction*' OR 'adverse' OR 'complications' OR 'toxicity' OR 'toxicities'))) OR TI=(('Stevens-Johnson Syndrome' OR 'SJS' OR 'toxic epidermal necrolysis' OR 'drug-induced hypersensitivity syndrome' OR 'DIHS' OR 'drug reaction with eosinophilia and systemic symptoms' OR 'DRESS' OR 'acute generalized exanthematous pustulosis' OR 'AGEP' OR 'exfoliative dermatitis' OR 'immunobullous disorder'))) AND TI=(('immune checkpoint inhibitor' OR 'immune checkpoint inhibitors' OR 'Immune checkpoint inhibition' OR 'immune checkpoint blockade*' OR 'immune checkpoint blocking' OR immunotherapy OR 'immune related' OR PD-1 OR 'programmed cell death protein 1' OR Pembrolizumab OR Keytruda OR Nivolumab OR Opdivo OR Cemiplimab OR Libtayo OR PD-L1 OR 'programmed death ligand 1' OR Atezolizumab OR Tecentriq OR Avelumab OR Bavencio OR Durvalumab OR Imfinzi OR 'CTLA 4' OR 'cytotoxic T lymphocyte associated protein 4' OR Ipilimumab OR Yervoy)) |

**Supplementary Table 2** Demographics and clinical characteristics of the patients with ICI-related SJS/TEN

| Patient(s) | First Author, Publication Year | Article Type | Quality Rating | Total Number of Patients With SJS/TEN | Classification | Age (y) | Sex | Cancer Type | Cancer Staging | PD-L1 Expression (%) | Concurrent Therapy | ICI Target | ICI Regimen | Other irAEs | ICI Cycles | Epidermal Detachment (% of BSA) |
| --- | --- | --- | --- | --- | --- | --- | --- | --- | --- | --- | --- | --- | --- | --- | --- | --- |
| 1 | Borg, 2022 (1) | case | 5 | 1 | TEN | 50 | M | Lung cancer | Ⅳ | NR | Carboplatin, pemetrexed | PD-1 | Pembrolizumab | N | 2 | 30 |
| 2 | Cai, 2020 (2) | case | 5 | 1 | TEN | 63 | M | Lung cancer | Ⅳ | 50 | Radiotherapy | PD-1 | Pembrolizumab | Pneumonitis | 1 | 40 |
| 3 | Chen, 2023 (3) | case | 4 | 1 | TEN | 60 | M | Hepatocellular carcinoma | NR | NR | Lenvatinib, transarterial chemoembolization | PD-1+CTLA-4 | Cadonilimab | N | 2 | 67 |
| 4 | Chirasuthat, 2018 (4) | case | 5 | 1 | SJS | 75 | M | Lung cancer | Ⅳ | NR | N | PD-L1 | Atezolizumab | N | 3 | 5 |
| 5 | Chow, 2022 (5) | case | 5 | 1 | TEN | 63 | M | Lung cancer | Ⅳ | 80 | Carboplatin, pemetrexed, radiotherapy | PD-1 | Pembrolizumab | N | 3 | 30 |
| 6 | Cui, 2020 (6) | case | 4 | 1 | TEN | 72 | F | Lung cancer | Ⅳ | 1 | Carboplatin, pemetrexed, osimertinib | PD-1 | Pembrolizumab | Esophagogastroenteritis | 2 | 90 |
| 7 | Gallo Marin, 2022 (7) | case | 5 | 1 | TEN | 77 | M | Gastrointestinal cancer | Ⅳ | NR | FOLFOX^a^, trastuzumab | PD-1 | Pembrolizumab | N | 2 | 90 |
| 8 | Goldinger, 2016 (8) | case | 4 | 1 | SJS | 77 | M | Melanoma | NR | NR | N | PD-1 | Pembrolizumab | N | 1 | NR |
| 9 | Gong, 2023 (9) | case | 4 | 1 | TEN | 78 | M | Intrahepatic cholangiocarcinoma | NR | NR | Lenvatinib | PD-1 | Sintilimab | Hepatitis | 1 | 90 |
| 10 | Gopee, 2020 (10) | case | 5 | 1 | TEN | 62 | M | Melanoma | Ⅲ | NR | N | PD-1+CTLA-4 | Nivolumab, ipilimumab | N | 3 | 30 |
| 11 | Gracia-Cazana, 2021 (11) | case | 5 | 1 | SJS | 78 | M | Lung cancer | Ⅲ | NR | N | PD-1 | Nivolumab | N | 2 | 12 |
| 12 | Griffin, 2018 (12) | case | 4 | 1 | TEN | 54 | M | Follicular lymphoma | NR | NR | Allopurinol, co-trimoxazole, fluconazole | PD-1 | Nivolumab | N | 1 | 90 |
| 13 | Haratake, 2018 (13) | case | 5 | 1 | SJS | 69 | M | Lung cancer | NR | NR | N | PD-1 | Pembrolizumab | N | 1 | 1 |
| 14 | Hsu, 2020 (14) | case series | 5 | 2 | SJS | 74 | F | Urothelial carcinoma | Ⅱ | NR | N | PD-1 | Pembrolizumab | N | 1 | 10 |
| 15 |  |  |  |  | TEN | 67 | F | Urothelial carcinoma | Ⅳ | NR | Isoniazid, ethambutol, rifampin, pyrazinamide | PD-L1 | Atezolizumab | N | 8 | 80 |
| 16 | Huang, 2022 (15) | case | 4 | 1 | TEN | 39 | M | Hepatocellular carcinoma | Ⅳ | NR | Lenvatinib | PD-1 | Toripalimab | N | 1 | 70 |
| 17 | Huang, 2022 (16) | case | 5 | 1 | SJS/TEN Overlap | 27 | F | Nasopharyngeal carcinoma | Ⅱ | NR | N | PD-1 | Sintilimab | N | 4 | NR |
| 18 | Ito, 2017 (17) | case | 5 | 1 | SJS | 76 | F | Lung cancer | Ⅳ | NR | N | PD-1 | Nivolumab | N | 2 | NR |
| 19 | Kian, 2022 (18) | case | 5 | 1 | TEN | 65 | M | Lung cancer | Ⅳ | 1 | N | PD-1 | Pembrolizumab | Esophagogastroenteritis, myocarditis | 1 | NR |
| 20 | Kim, 2021 (19) | case | 5 | 1 | TEN | 86 | M | Hepatocellular carcinoma | NR | NR | N | PD-1 | Nivolumab | N | 2 | 30 |
| 21 | Konstantina, 2019 (20) | case | 5 | 1 | SJS | 58 | F | Thymoma | Ⅲ | 80 | N | PD-1 | Pembrolizumab | Hepatitis, myocarditis | 1 | NR |
| 22 | Koshizuka, 2021 (21) | case | 5 | 1 | TEN | 76 | M | Tongue cancer | Ⅳ | NR | N | PD-1 | Nivolumab | N | 2 | NR |
| 23 | Kubicki, 2018 (22) | case | 5 | 1 | TEN | 47 | M | Gastrointestinal cancer | Ⅳ | NR | N | PD-1+CTLA-4 | Nivolumab, ipilimumab | N | 1 | 54 |
| 24 | Kumar, 2020 (23) | case | 4 | 1 | TEN | 57 | F | Lung cancer | Ⅳ | 10 | N | PD-1 | Pembrolizumab | N | 1 | 80 |
| 25 | Li, 2022 (24) | case | 5 | 1 | TEN | 59 | M | Lung cancer | Ⅱ | NR | Paclitaxel, cisplatin | PD-1 | Sintilimab | N | 4 | 95 |
| 26 | Li, 2023 (25) | case | 5 | 1 | SJS | 76 | M | Lung cancer | Ⅲ | 1 | Paclitaxel liposome | PD-1 | Sintilimab | N | 3 | NR |
| 27 | Logan, 2020 (26) | case | 5 | 1 | TEN | 62 | M | Melanoma | Ⅳ | NR | N | PD-1+CTLA-4 | Nivolumab, ipilimumab | N | 2 | 80 |
| 28 | Lye, 2023 (27) | case | 5 | 1 | TEN | 72 | M | Lung cancer | Ⅳ | NR | Paclitaxel, carboplatin | PD-1 | Sintilimab | N | 1 | NR |
| 29 | Nayar, 2016 (28) | case | 5 | 1 | TEN | 64 | F | Melanoma | Ⅳ | NR | N | PD-1 | Nivolumab | N | 2 | NR |
| 30 | Oguri, 2021 (29) | case | 5 | 1 | SJS/TEN Overlap | 76 | M | Lung cancer | Ⅳ | 90 | Denosumab, radiotherapy | PD-1 | Pembrolizumab | Guillain-Barré syndrome | 1 | 18 |
| 31 | Otero, 2023 (30) | case | 5 | 1 | SJS | 65 | F | Keratinizing squamous cell vulvar carcinoma | Ⅳ | NR | N | PD-1 | Nivolumab | N | 2 | NR |
| 32 | Pathria, 2016 (31) | case | 5 | 1 | SJS/TEN Overlap | 71 | F | Melanoma | Ⅳ | NR | N | CTLA-4 | Ipilimumab | N | 3 | 25 |
| 33 | Potts, 2022 (32) | case | 5 | 1 | SJS | 80 | F | Renal cell carcinoma | Ⅳ | NR | N | PD-1 | Nivolumab | N | 3 | NR |
| 34 | Robinson, 2020 (33) | case | 5 | 1 | SJS | 55 | F | Cervical squamous cell carcinoma | Ⅳ | NR | N | PD-1 | Pembrolizumab | N | 1 | NR |
| 35 | Ryu, 2022 (34) | case | 5 | 1 | SJS | 64 | M | Urothelial carcinoma | Ⅳ | NR | N | PD-1 | Pembrolizumab | N | 1 | NR |
| 36 | Saad, 2022 (35) | case | 5 | 1 | SJS/TEN Overlap | 45 | F | Gastrointestinal cancer | Ⅳ | NR | FOLFOX, radiotherapy | PD-1 | Nivolumab | N | 3 | 15 |
| 37 | Salati, 2018 (36) | case | 5 | 1 | SJS | 59 | F | Lung cancer | Ⅳ | NR | N | PD-1 | Nivolumab | N | 2 | NR |
| 38 | Sandhu, 2023 (37) | case | 5 | 1 | SJS | 75 | F | Lung cancer | Ⅳ | NR | N | PD-1 | Pembrolizumab | N | 1 | NR |
| 39 | Saw, 2017 (38) | case series | 5 | 2 | SJS | 50 | F | Nasopharyngeal carcinoma | Ⅳ | NR | N | PD-1 | Pembrolizumab | N | 5 | 5 |
| 40 |  |  |  |  | SJS | 53 | M | Renal cell carcinoma | Ⅳ | NR | Radiotherapy | PD-1 | Pembrolizumab | N | 3 | 3 |
| 41 | Sommerfelt, 2022 (39) | case series | 4 | 2 | TEN | 54 | F | Melanoma | Ⅳ | NR | Co-trimoxazole | PD-1+CTLA-4 | Nivolumab, ipilimumab | Hypophysitis, hepatitis | NR | 90 |
| 42 |  |  |  |  | TEN | 34 | M | Renal cell carcinoma | Ⅳ | NR | Pivmecillinam, piperacillin/tazobactam | PD-1+CTLA-4 | Nivolumab, ipilimumab | N | 1 | 90 |
| 43 | Vivar, 2017 (40) | case | 5 | 1 | TEN | 50 | F | Melanoma | Ⅳ | NR | N | PD-1 | Nivolumab | N | 3 | NR |
| 44 | Watanabe, 2020 (41) | case | 5 | 1 | TEN | 60 | F | Melanoma | Ⅳ | NR | N | PD-1 | Nivolumab | Hashimoto disease | NR | 30 |
| 45 | Wu, 2022 (42) | case | 4 | 1 | SJS | 68 | F | Lung cancer | Ⅳ | NR | Acetaminophen, famotidine, antibiotics | PD-1 | Pembrolizumab | N | 1 | 30 |
| 46 | Yang, 2022 (43) | case | 5 | 1 | TEN | 82 | M | Thymoma | Ⅳ | NR | Paclitaxel, cisplatin | PD-1 | Sintilimab | N | 2 | NR |
| 47 | Zhang, 2020 (44) | case | 5 | 1 | TEN | 34 | F | Gastrointestinal cancer | Ⅳ | NR | N | PD-1 | Pembrolizumab | N | 2 | NR |
| 48 | Zhang, 2022 (45) | case | 5 | 1 | SJS | 32 | M | Hepatocellular carcinoma | Ⅳ | NR | Paclitaxel, tegafur | PD-1 | Pembrolizumab | N | 3 | 8 |
| 49 | Zhang, 2023 (46) | case | 5 | 1 | TEN | 70 | F | Gastrointestinal cancer | Ⅳ | NR | N | PD-1 | Sintilimab | N | 1 | 70 |
| 50 | Zhao, 2022 (47) | case | 5 | 1 | TEN | 72 | F | Urothelial carcinoma | Ⅳ | NR | Oxaliplatin, tiggio | PD-1 | Sintilimab | N | 1 | NR |

Abbreviations: BSA, body surface area; CTLA-4, cytotoxic T lymphocyte-associated antigen 4; F, female; ICI, immune checkpoint inhibitor; M, male; NR, not reported; PD-1, programmed cell death protein-1; PD-L1, programmed cell death-ligand 1; SJS, Stevens-Johnson syndrome; TEN, toxic epidermal necrolysis.

^a^ FOLFOX includes oxaliplatin, 5-fluorouracil, and leucovorin.

**Supplementary Table 3** Clinical manifestations of the patients with ICI-related SJS/TEN

| Patient(s) | First Author, Publication Year | Time from ICI Initiation to Onset (d) | Preceding Rashes | Fever | Oral Involvement | Ocular Involvement | Genital Involvement | Histopathologic Findings consistent with SJS/TEN | SCORTEN |
| --- | --- | --- | --- | --- | --- | --- | --- | --- | --- |
| 1 | Borg, 2022 (1) | 24 | N | NR | N | N | N | Y | 3 |
| 2 | Cai, 2020 (2) | 3 | N | NR | Y | Y | Y | Y | 3 |
| 3 | Chen, 2023 (3) | 24 | N | NR | NR | NR | NR | N | 5 |
| 4 | Chirasuthat, 2018 (4) | 60 | Rash, oral mucositis | NR | Y | Y | N | Y | NR |
| 5 | Chow, 2022 (5) | 66 | N | NR | Y | N | Y | Y | 3 |
| 6 | Cui, 2020 (6) | 63 | N | Y | Y | Y | N | Y | 3 |
| 7 | Gallo Marin, 2022 (7) | 22 | N | Y | N | N | N | Y | 3 |
| 8 | Goldinger, 2016 (8) | 7 | N | NR | Y | NR | Y | Y | 3 |
| 9 | Gong, 2023 (9) | 18 | N | Y | Y | Y | Y | NR | 3 |
| 10 | Gopee, 2020 (10) | 63 | Maculopapular rash | N | Y | N | N | Y | 3 |
| 11 | Gracia-Cazana, 2021 (11) | 30 | N | Y | Y | Y | NR | Y | 3 |
| 12 | Griffin, 2018 (12) | 10 | N | NR | NR | Y | NR | Y | 3 |
| 13 | Haratake, 2018 (13) | 12 | N | Y | Y | Y | N | Y | NR |
| 14 | Hsu, 2020 (14) | 10 | N | NR | Y | N | Y | Y | NR |
| 15 |  | 195 | N | NR | Y | Y | Y | Y | NR |
| 16 | Huang, 2022 (15) | 21 | N | Y | Y | Y | Y | Y | NR |
| 17 | Huang, 2022 (16) | 70 | N | Y | Y | Y | N | Y | NR |
| 18 | Ito, 2017 (17) | 14 | N | Y | NR | Y | NR | Y | NR |
| 19 | Kian, 2022 (18) | 3 | N | NR | Y | NR | NR | Y | NR |
| 20 | Kim, 2021 (19) | 28 | N | N | Y | N | N | Y | 3 |
| 21 | Konstantina, 2019 (20) | 7 | N | Y | Y | NR | NR | Y | NR |
| 22 | Koshizuka, 2021 (21) | 26 | N | Y | N | N | N | Y | NR |
| 23 | Kubicki, 2018 (22) | 6 | N | Y | Y | Y | Y | Y | 3 |
| 24 | Kumar, 2020 (23) | 14 | N | NR | Y | Y | N | NR | NR |
| 25 | Li, 2022 (24) | 149 | N | Y | Y | NR | NR | NR | NR |
| 26 | Li, 2023 (25) | 60 | N | NR | Y | N | N | Y | NR |
| 27 | Logan, 2020 (26) | 25 | Grade 1 rash | NR | Y | N | N | Y | 5 |
| 28 | Lye, 2023 (27) | 17 | N | NR | Y | N | Y | NR | 3 |
| 29 | Nayar, 2016 (28) | 28 | N | NR | N | N | N | Y | NR |
| 30 | Oguri, 2021 (29) | 23 | N | NR | Y | N | N | Y | NR |
| 31 | Otero, 2023 (30) | 23 | N | NR | Y | N | Y | Y | NR |
| 32 | Pathria, 2016 (31) | 18 | N | NR | Y | Y | Y | Y | NR |
| 33 | Potts, 2022 (32) | 270 | Lichenoid dermatitis | NR | Y | Y | N | Y | NR |
| 34 | Robinson, 2020 (33) | 17 | N | Y | Y | Y | N | Y | NR |
| 35 | Ryu, 2022 (34) | 4 | N | NR | N | Y | N | N | NR |
| 36 | Saad, 2022 (35) | 56 | N | N | Y | N | N | Y | 4 |
| 37 | Salati, 2018 (36) | 21 | N | Y | Y | NR | N | NR | NR |
| 38 | Sandhu, 2023 (37) | 14 | N | NR | Y | Y | NR | Y | NR |
| 39 | Saw, 2017 (38) | 140 | N | NR | Y | Y | N | Y | NR |
| 40 |  | 77 | N | NR | Y | Y | N | Y | NR |
| 41 | Sommerfelt, 2022 (39) | 120 | N | NR | Y | Y | Y | Y | 3 |
| 42 |  | 5 | N | NR | Y | Y | Y | Y | 3 |
| 43 | Vivar, 2017 (40) | 42 | Maculopapular rash | N | Y | Y | Y | Y | 5 |
| 44 | Watanabe, 2020 (41) | 210 | N | Y | Y | Y | N | Y | 2 |
| 45 | Wu, 2022 (42) | 21 | N | Y | Y | Y | Y | NR | 4 |
| 46 | Yang, 2022 (43) | 21 | Maculopapular rash | NR | Y | N | N | Y | 7 |
| 47 | Zhang, 2020 (44) | 60 | N | NR | N | N | N | NR | NR |
| 48 | Zhang, 2022 (45) | 105 | N | NR | Y | N | N | Y | NR |
| 49 | Zhang, 2023 (46) | 10 | N | NR | NR | NR | NR | NR | 5 |
| 50 | Zhao, 2022 (47) | 7 | N | Y | NR | NR | NR | Y | NR |

Abbreviations: ICI, immune checkpoint inhibitor; N, no; NR, not reported; SCORTEN, severity-of-illness score for toxic epidermal necrolysis; SJS, Stevens-Johnson syndrome; TEN, toxic epidermal necrolysis; Y, yes.

**Supplementary Table 4** Management and outcomes of the patients with ICI-related SJS/TEN

| Patient(s) | First Author, Publication Year | Systemic CS | CS Dose (mg/kg/day) | CS Duration (d) | IVIG | IVIG Cumulative Dose (g/kg) | CsA | CsA Dose (mg/kg/day) | CsA Duration (d) | TNF-α inhibitors | Other Treatments | SJS/TEN Outcome | Time to re-epithelization (d) | Objective tumor response | Cause of Death |
| --- | --- | --- | --- | --- | --- | --- | --- | --- | --- | --- | --- | --- | --- | --- | --- |
| 1 | Borg, 2022 (1) | N | NR | NR | Y | 3 | Y | 5 | NR | NR | NR | Recovered | NR | SD | Alive |
| 2 | Cai, 2020 (2) | Y | 3.2 | 21 | NR | NR | Y | 6 | 14 | NR | NR | Recovered | 25 | SD | Alive |
| 3 | Chen, 2023 (3) | Y | 5 | 63 | Y | 2 | NR | NR | NR | Adalimumab 80 mg once | NR | Recovered | 24 | NR | Alive |
| 4 | Chirasuthat, 2018 (4) | Y | NR | 15 | NR | NR | NR | NR | NR | NR | NR | Recovered | 14 | SD | Alive |
| 5 | Chow, 2022 (5) | Y | 3.75 | 30 | Y | 2 | NR | NR | 7 | NR | NR | Recovered | NR | PD | Cancer progression |
| 6 | Cui, 2020 (6) | Y | 2.5 | NR | Y | 2 | NR | NR | NR | NR | NR | Died | Died | NR | NR |
| 7 | Gallo Marin, 2022 (7) | Y | 2 | 63 | NR | NR | Y | 4 | 21 | NR | NR | Recovered | 34 | SD | Alive |
| 8 | Goldinger, 2016 (8) | Y | 1 | 28 | NR | NR | NR | NR | NR | NR | NR | Recovered | NR | NR | Alive |
| 9 | Gong, 2023 (9) | Y | 2.63 | NR | Y | 2.5 | NR | NR | NR | NR | NR | Died | Died | NR | Hypovolemic |
| 10 | Gopee, 2020 (10) | Y | 2.5 | 28 | NR | NR | NR | NR | NR | NR | NR | Recovered | 28 | SD | Alive |
| 11 | Gracia-Cazana, 2021 (11) | Y | NR | 30 | NR | NR | NR | NR | NR | NR | NR | Recovered | 28 | PD | Cancer progression |
| 12 | Griffin, 2018 (12) | Y | NR | NR | Y | NR | NR | NR | NR | NR | MP 1g 3d | Died | Died | NR | Infection |
| 13 | Haratake, 2018 (13) | Y | NR | 30 | NR | NR | NR | NR | NR | NR | NR | Recovered | 30 | NR | Alive |
| 14 | Hsu, 2020 (14) | Y | NR | NR | NR | NR | NR | NR | NR | NR | NR | Recovered | NR | PD | Cancer progression |
| 15 |  | Y | NR | 9 | NR | NR | NR | NR | NR | NR | NR | Recovered | NR | PR | Alive |
| 16 | Huang, 2022 (15) | Y | NR | 60 | Y | 2 | NR | NR | NR | NR | NR | Recovered | NR | SD | Alive |
| 17 | Huang, 2022 (16) | Y | NR | NR | Y | 2 | NR | NR | NR | NR | NR | Recovered | NR | NR | Alive |
| 18 | Ito, 2017 (17) | Y | 1 | 27 | NR | NR | NR | NR | NR | NR | NR | Recovered | NR | PD | Alive |
| 19 | Kian, 2022 (18) | Y | 2 | 19 | Y | 2 | NR | NR | NR | NR | MP 0.5g 3d | Recovered | 24 | NR | Alive |
| 20 | Kim, 2021 (19) | Y | NR | NR | Y | NR | NR | NR | NR | NR | NR | Died | Died | PD | Infection |
| 21 | Konstantina, 2019 (20) | Y | 1 | NR | NR | NR | NR | NR | NR | NR | Mycophenolate mofetil | Died | Died | PD | heart failure |
| 22 | Koshizuka, 2021 (21) | Y | NR | 39 | Y | 2 | NR | NR | NR | NR | NR | Died | Died | PD | Cancer progression |
| 23 | Kubicki, 2018 (22) | Y | 2 | 51 | NR | NR | NR | NR | NR | NR | NR | Recovered | 51 | NR | Alive |
| 24 | Kumar, 2020 (23) | Y | 1 | NR | NR | NR | NR | NR | NR | Infliximab 5 mg/kg once | Plasmapheresis | Died | Died | NR | NR |
| 25 | Li, 2022 (24) | Y | NR | NR | NR | NR | NR | NR | NR | NR | NR | Recovered | 71 | NR | Alive |
| 26 | Li, 2023 (25) | Y | NR | 53 | Y | NR | NR | NR | NR | NR | NR | Recovered | NR | PD | Alive |
| 27 | Logan, 2020 (26) | N | NR | NR | Y | 8 | Y | 3 | 14 | NR | G-CSF | Died | Died | PD | Cancer progression |
| 28 | Lye, 2023 (27) | Y | NR | 25 | NR | NR | NR | NR | NR | NR | NR | Recovered | NR | NR | Alive |
| 29 | Nayar, 2016 (28) | Y | 1.875 | NR | Y | NR | Y | 3 | NR | NR | NR | Recovered | NR | PD | Infection |
| 30 | Oguri, 2021 (29) | Y | 1 | NR | Y | 2 | NR | NR | NR | NR | NR | Recovered | 33 | PD | Infection |
| 31 | Otero, 2023 (30) | N | NR | 28 | NR | NR | NR | NR | NR | NR | NR | Recovered | 28 | PR | Alive |
| 32 | Pathria, 2016 (31) | Y | NR | NR | NR | NR | NR | NR | NR | NR | NR | Recovered | NR | NR | Alive |
| 33 | Potts, 2022 (32) | Y | NR | 30 | Y | NR | NR | NR | NR | NR | NR | Recovered | 30 | NR | Alive |
| 34 | Robinson, 2020 (33) | Y | NR | NR | NR | NR | NR | NR | NR | NR | NR | Recovered | 30 | PD | Alive |
| 35 | Ryu, 2022 (34) | Y | NR | 90 | NR | NR | NR | NR | NR | NR | NR | Recovered | NR | NR | Alive |
| 36 | Saad, 2022 (35) | Y | 1 | NR | NR | NR | NR | NR | NR | NR | NR | Recovered | NR | NR | Alive |
| 37 | Salati, 2018 (36) | Y | 1.25 | NR | NR | NR | NR | NR | NR | NR | NR | Recovered | NR | NR | Alive |
| 38 | Sandhu, 2023 (37) | Y | NR | 30 | Y | 3 | NR | NR | NR | NR | NR | Recovered | NR | NR | Alive |
| 39 | Saw, 2017 (38) | Y | NR | 4 | NR | NR | Y | 3 | 20 | NR | NR | Recovered | 14 | SD | Alive |
| 40 |  | N | NR | NR | NR | NR | Y | 3.78 | 22 | NR | NR | Recovered | 21 | PR | Alive |
| 41 | Sommerfelt, 2022 (39) | Y | NR | NR | Y | NR | Y | 3.2 | NR | NR | NR | Recovered | 39 | CR | Alive |
| 42 |  | Y | NR | NR | NR | NR | Y | 4.5 | NR | NR | Mycophenolate mofetil | Died | Died | PD | Cancer progression |
| 43 | Vivar, 2017 (40) | Y | 1 | NR | Y | 3 | NR | NR | NR | Infliximab 5mg/kg once | NR | Died | Died | NR | Infection |
| 44 | Watanabe, 2020 (41) | Y | NR | 28 | Y | 2 | NR | NR | NR | NR | Plasmapheresis | Recovered | 18 | PD | Cancer progression |
| 45 | Wu, 2022 (42) | Y | 2.5 | 90 | Y | NR | NR | NR | NR | Etanercept 25mg twice a week | NR | Recovered | NR | SD | Alive |
| 46 | Yang, 2022 (43) | Y | 1.875 | 7 | Y | NR | NR | NR | NR | NR | NR | Recovered | 30 | NR | Pneumonia |
| 47 | Zhang, 2020 (44) | Y | 1.25 | 56 | Y | 1 | NR | NR | NR | NR | NR | Recovered | 16 | NR | Alive |
| 48 | Zhang, 2022 (45) | Y | 1.25 | 33 | NR | NR | NR | NR | NR | Adalimumab 80mg once | NR | Recovered | 30 | PD | Cancer progression |
| 49 | Zhang, 2023 (46) | Y | NR | NR | Y | 1.2 | NR | NR | NR | Adalimumab 40mg twice | NR | Recovered | 45 | NR | Alive |
| 50 | Zhao, 2022 (47) | Y | 2 | NR | Y | NR | NR | NR | NR | NR | NR | Recovered | 43 | NR | Alive |

Abbreviations: CR, complete response; CS, corticosteroid; CsA, Cyclosporine; ICI, immune checkpoint inhibitor; IVIG, intravenous immunoglobulins; MP, Methylprednisolone; N, no; NR, not reported; PD, progressive disease; PR, partial response; SD, stable disease; SJS, Stevens-Johnson syndrome; TEN, toxic epidermal necrolysis; TNF-α, tumor necrosis factor-α; Y, yes.

**References**

1. Borg L, Buhagiar M, La Ferla E, Pisani D, Said J, Boffa MJ. Pembrolizumab-Induced Toxic Epidermal Necrolysis. Case Reports in Oncology. 2022;15(3):887-93.

2. Cai ZR, Lecours J, Adam JP, Marcil I, Blais N, Dallaire M, et al. Toxic epidermal necrolysis associated with pembrolizumab. Journal of Oncology Pharmacy Practice. 2020;26(5):1259-65.

3. Chen PY, Li ZY, Cai SQ. Case Report: Cadonilimab-related toxic epidermal necrolysis-like reactions successfully treated with supplemental Adalimumab. Front Immunol. 2023;14:1188523.

4. Chirasuthat P, Chayavichitsilp P. Atezolizumab-Induced Stevens-Johnson Syndrome in a Patient with Non-Small Cell Lung Carcinoma. Case Reports in Dermatology. 2018;10(2):198-202.

5. Chow KVC, O'Leary C, Paxton-Hall F, Lambie D, O'Byrne K. Pembrolizumab-induced toxic epidermal necrolysis: Case report. Oxford Medical Case Reports. 2022;2022(3):112-4.

6. Cui W, Cotter C, Sreter KB, Heelan K, Creamer D, Basu TN, et al. Case of fatal immune-related skin toxicity from sequential use of osimertinib after pembrolizumab: Lessons for drug sequencing in never-smoking non–Small-cell lung cancer. JCO Oncology Practice. 2020;16(12):842-4.

7. Gallo Marin B, Oliva R, Kahn B, Borgovan T, Brooks BE, Massoud CM. Pembrolizumab-induced Toxic Epidermal Necrolysis in a Patient with Metastatic Esophageal Adenocarcinoma. R I Med J (2013). 2022;105(3):34-6.

8. Goldinger SM, Stieger P, Meier B, Micaletto S, Contassot E, French LE, et al. Cytotoxic cutaneous adverse drug reactions during anti-PD-1 therapy. Clinical Cancer Research. 2016;22(16):4023-9.

9. Gong Y, Mao J, Liu M, Gao J. A case of toxic epidermal necrolysis associated with lenvatinib and sintilimab therapy for intrahepatic cholangiocarcinoma. Journal of International Medical Research. 2023;51(5):3000605231173556.

10. Gopee NH, Gourley AM, Oliphant TJ, Hampton PJ. Toxic epidermal necrolysis occurring with immune checkpoint inhibitors. Dermatology Online Journal. 2020;26(8):13030/qt8fc428f6.

11. Gracia-Cazaña T, Padgett E, Calderero V, Oncins R. Nivolumab-associated Stevens-Johnson syndrome in a patient with lung cancer. Dermatology Online Journal. 2021;27(3):13030/qt2897t6dq.

12. Griffin LL, Cove-Smith L, Alachkar H, Radford JA, Brooke R, Linton KM. Toxic epidermal necrolysis (TEN) associated with the use of nivolumab (PD-1 inhibitor) for lymphoma. JAAD Case Reports. 2018;4(3):229-31.

13. Haratake N, Tagawa T, Hirai F, Toyokawa G, Miyazaki R, Maehara Y. Stevens-Johnson Syndrome Induced by Pembrolizumab in a Lung Cancer Patient. Journal of Thoracic Oncology. 2018;13(11):1798-9.

14. Hsu T-J, Liu K-L. Stevens-Johnson syndrome and toxic epidermal necrolysis related to immune checkpoint inhibitors: Two cases and literature review. DERMATOLOGICA SINICA. 2020;38(4):236-9.

15. Huang KK, Han SS, He LY, Yang LL, Liang BY, Zhen QY, et al. Combination therapy (toripalimab and lenvatinib)-associated toxic epidermal necrolysis in a patient with metastatic liver cancer: A case report. World Journal of Clinical Cases. 2022;10(11):3478-84.

16. Huang Y, Zhu L, Ma X, Hong Y, Su X, Lai W, et al. A case of sintilimab-induced SJS/TEN:Dermatologic adverse reactions associated with programmed cell death protein-1 inhibitors. DERMATOLOGIC THERAPY. 2022;35(9):e15663.

17. Ito J, Fujimoto D, Nakamura A, Nagano T, Uehara K, Imai Y, et al. Aprepitant for refractory nivolumab-induced pruritus. Lung Cancer. 2017;109:58-61.

18. Kian W, Zemel M, Elobra F, Sharb AA, Levitas D, Assabag Y, et al. Intravenous immunoglobulin efficacy on pembrolizumab induced severe toxic epidermal necrolysis. Anti-Cancer Drugs. 2022;33(1):E738-E40.

19. Kim MC, Khan HN. Nivolumab-Induced Toxic Epidermal Necrolysis: Rare but Fatal Complication of Immune Checkpoint Inhibitor Therapy. Cureus. 2021;13(5):e15017.

20. Konstantina T, Konstantinos R, Anastasios K, Anastasia M, Eleni L, Ioannis S, et al. Fatal adverse events in two thymoma patients treated with anti-PD-1 immune check point inhibitor and literature review. Lung Cancer. 2019;135:29-32.

21. Koshizuka K, Sakurai D, Sunagane M, Mita Y, Hamasaki S, Suzuki T, et al. Toxic epidermal necrolysis associated with nivolumab treatment for head and neck cancer. Clinical Case Reports. 2021;9(2):848-52.

22. Kubicki SL WM, Patel AB. . Toxic epidermal necrolysis during cotherapy with ipilimumab and nivolumab. J Immunother Precis Oncol. 2018;1:78-81.

23. Kumar R, Bhandari S. Pembrolizumab induced toxic epidermal necrolysis. CURRENT PROBLEMS IN CANCER. 2020;44(2):100478.

24. Li G, Gong S, Wang N, Yao X. Toxic epidermal necrolysis induced by sintilimab in a patient with advanced non-small cell lung cancer and comorbid pulmonary tuberculosis: A case report. Frontiers in Immunology. 2022;13:989966.

25. Li X, Li G, Chen D, Su L, Wang RP, Zhou Y. Case Report: sintilimab-induced Stevens-Johnson Syndrome in a patient with advanced lung adenocarcinoma. Frontiers in Oncology. 2023;13:912168.

26. Logan IT, Zaman S, Hussein L, Perrett CM. Combination Therapy of Ipilimumab and Nivolumab-associated Toxic Epidermal Necrolysis (TEN) in a Patient With Metastatic Melanoma: A Case Report and Literature Review. JOURNAL OF IMMUNOTHERAPY. 2020;43(3):89-92.

27. Lye YL, Shan B, Jia CH, Liu J, Hou J, Du WL, et al. Toxic Epidermal Necrolysis Induced by Sintilimab: A Case Report. Annals of Dermatology. 2023;35:S100-S2.

28. Nayar N, Briscoe K, Penas PF. Toxic epidermal necrolysis-like reaction with severe satellite cell necrosis associated with nivolumab in a patient with ipilimumab refractory metastatic melanoma. Journal of Immunotherapy. 2016;39(3):149-52.

29. Oguri T, Sasada S, Shimizu S, Shigematsu R, Tsuchiya Y, Ishioka K, et al. A Case of Guillain-Barré Syndrome and Stevens-Johnson Syndrome/Toxic Epidermal Necrosis Overlap After Pembrolizumab Treatment. Journal of Investigative Medicine High Impact Case Reports. 2021;9:23247096211037462.

30. Rodríguez-Otero N, Chamorro-Pérez J, Fernández-Lozano C, Elías-Sáenz I, Berná-Rico E, de Nicolás-Ruanes B, et al. Nivolumab-induced Stevens-Johnson syndrome: Not only due to PD-1 inhibition. Journal of Allergy and Clinical Immunology: In Practice. 2023;11(9):2936-8.e1.

31. Pathria M MJ, Trufant J. A case of Stevens–Johnson syndrome in a patient on ipilimumab. Int J Case Rep Imag. 2016;7(5):300–2.

32. Potts J, Lee RR, Hilliard CA. Lichenoid dermatitis preceding Stevens-Johnson syndrome in a patient treated with nivolumab. BMJ Case Reports. 2022;15(8):e251233.

33. Robinson S, Saleh J, Curry J, Mudaliar K. Pembrolizumab-Induced Stevens-Johnson Syndrome/Toxic Epidermal Necrolysis in a Patient with Metastatic Cervical Squamous Cell Carcinoma: A Case Report. American Journal of Dermatopathology. 2020;42(4):292-6.

34. Ryu S, Jun I, Kim T-I, Seo KY, Kim EK. Pembrolizumab-induced Stevens-Johnson Syndrome with Severe Ocular Complications. Ocular immunology and inflammation. 2022;30(6):1533-5.

35. Saad E, Adhikari P, Antala D, Abdulrahman A, Begiashvili V, Mohamed K, et al. Steven-Johnson Syndrome: A Rare but Serious Adverse Event of Nivolumab Use in a Patient With Metastatic Gastric Adenocarcinoma. J Med Cases. 2022;13(9):449-55.

36. Salati M, Pifferi M, Baldessari C, Bertolini F, Tomasello C, Cascinu S, et al. Stevens-Johnson syndrome during nivolumab treatment of NSCLC. ANNALS OF ONCOLOGY. 2018;29(1):283-4.

37. Sandhu M, Kc B, Bhandari J, Gambhir HS, Farah R. Pembrolizumab-Associated Stevens-Johnson Syndrome in a Patient With Metastatic Non-small Cell Lung Cancer: A Case Report. Cureus. 2023;15(7):e41439.

38. Saw S, Lee HY, Ng QS. Pembrolizumab-induced Stevens-Johnson syndrome in non-melanoma patients. EUROPEAN JOURNAL OF CANCER. 2017;81:237-9.

39. Sommerfelt H, Sandvik LF, Bachmann IM, Brekke RL, Svendsen HL, Guttormsen AB, et al. Toxic epidermal necrolysis after immune checkpoint inhibition, case report, and review of the literature. ACTA ONCOLOGICA. 2022;61(10):1295-9.

40. Vivar KL, Deschaine M, Messina J, Divine JM, Rabionet A, Patel N, et al. Epidermal programmed cell death-ligand 1 expression in TEN associated with nivolumab therapy. Journal of cutaneous pathology. 2017;44(4):381-4.

41. Watanabe Y, Yamaguchi Y, Takamura N, Takahashi Y, Aihara M. Toxic epidermal necrolysis accompanied by several immune-related adverse events developed after discontinuation of nivolumab. EUROPEAN JOURNAL OF CANCER. 2020;131:1-4.

42. Wu JY, Kang K, Yi J, Yang B. Pembrolizumab-induced Stevens-Johnson syndrome in advanced squamous cell carcinoma of the lung: A case report and review of literature. World Journal of Clinical Cases. 2022;10(18):6110-8.

43. Yang H, Ma Q, Sun Y, Zhang K, Xing Y, Li H. Case Report: Toxic epidermal necrolysis associated with sintilimab in a patient with relapsed thymic carcinoma. Frontiers in Oncology. 2022;12:1065137.

44. Zhang L, Shen L, Lu Y, Xue J. Cancer immunotherapy and toxic epidermal necrolysis. BMJ SUPPORTIVE & PALLIATIVE CARE. 2020;10(3):314-5.

45. Zhang J, Zhang P, Xu Q-Y, Zhu Y-T, Chen W, Ji C. Pembrolizumab associated Stevens-Johnson syndrome with porokeratosis in a patient in the setting of primary hepatocellular carcinoma. AUSTRALASIAN JOURNAL OF DERMATOLOGY. 2022;63(1):E71-E4.

46. Zhang L, Wu Z. Adalimumab for Sintilimab-Induced Toxic Epidermal Necrolysis in a Patient with Metastatic Gastric Malignancy: A Case Report and Literature Review. Clinical, Cosmetic and Investigational Dermatology. 2023;16:457-61.

47. Zhao Y, Cao Y, Wang X, Qian T. Treatment of PD-1 Inhibitor-Associated Toxic Epidermal Necrolysis: A Case Report and Brief Review. OncoTargets and Therapy. 2022;15:345-51.
